# Efficacy of traditional Chinese medicine external therapy on cancer-related fatigue: a systematic review and network meta-analysis

**DOI:** 10.3389/fonc.2026.1806355

**Published:** 2026-04-22

**Authors:** Xinyu Wang, Shujuan Chen, Fanjiao Meng, Wei Zhou, Yihong Jiang, Jinhong Yang

**Affiliations:** 1 School of Nursing, Shandong Second Medical University, Weifang, Shandong, China; 2 Department of Oncology, Weifang People’s Hospital (The First Affiliated Hospital of Shandong Second Medical University), Weifang, Shandong, China

**Keywords:** acupuncture, cancer patients, cancer-related fatigue, network meta-analysis, traditional Chinese medicine

## Abstract

**Objectives:**

The purpose of this network meta-analysis was to compare and evaluate the effectiveness of several traditional Chinese medicine (TCM) external therapies in reducing cancer-related fatigue(CRF).

**Methods:**

A search of eight databases was conducted to obtain randomized controlled trials (RCTs) on different external therapies of traditional Chinese medicine for alleviating CRF. The Cochrane ROB 2.0 tool was used to assess the included studies’ bias risk. Network meta-analysis and comparative effect ranking were conducted using STATA MP 17.0. The primary outcome was the CRF score. Standardized mean difference (SMD) and 95% confidence interval (95% CI) were used to calculate the effect size. The Confidence in Network Meta-Analysis (CINeMA) tool was used to assess the certainty of the evidence.

**Results:**

The systematic review included eighty-seven RCTs covering 13 different external therapies. Among the treatments studied, acupuncture, acupressure, traditional Chinese exercises, transcutaneous acupoint electrical stimulation, moxibustion, Chinese medicine foot bath, traditional Chinese medicine (TCM)emotional care, acupoint injection, acupoint application, warming needle were demonstrated to be effective in alleviating CRF in comparison to usual care. The surface under cumulative ranking curve (SUCRA) indicated that warming needle was most effective in relieving CRF, followed by Chinese medicine foot bath, TCM emotional care, acupoint injections, moxibustion, etc.

**Conclusions:**

This review indicated that warming needle might be the most effective traditional Chinese medicine external therapy for treating CRF. But, the low certainty of evidence (assessed via CINeMA) must be emphasized in this low confidence means the results should be interpreted with great caution. Further validation is required through additional clinical trials. These findings provide significant support for future research and clinical trials.

**Systematic Review Registration:**

https://www.crd.york.ac.uk/prospero/, identifier CRD42023479194.

## 1 Introduction

Cancer is a major global public health issue, with approximately 20 million new cases reported in 2022 and projections suggesting an increase to 35 million by 2050, according to the International Agency for Research on Cancer (IARC) (1). Advances in medical treatments have steadily increased the number of cancer survivors, generating a growing interest in improving their quality of life (2). Cancer and its treatment can cause numerous adverse symptoms for patients and survivors, with cancer-related fatigue (CRF) among the most common and persistent (3). CRF affects about 40% of patients at diagnosis (4), with incidence rates during treatment reaching 62-85% and 30-60% of cases being moderate to severe (5, 6). Long-term follow-ups indicate that severe CRF affects approximately 30% of survivors (7).

CRF is a persistent, subjective experience of physical, emotional, or cognitive fatigue related to cancer or its treatment, and it is notably more intense than typical fatigue (8). Unlike ordinary fatigue, CRF is not relieved by rest and disrupts daily living, impacting work, social relationships, and mood, and diminishing quality of life (9–12). Additionally, CRF can reduce patients’ tolerance to chemotherapy, complicate treatment adherence, and potentially lead to therapy cessation, all of which can negatively impact survival rates (13).

Management of CRF involves both pharmacological and non-pharmacological approaches (14). Commonly used drugs, such as psychiatric stimulants and hormones, have not shown significant benefits for CRF in randomized trials (15–17). Moreover, medication adherence is low, with around half of patients with chronic conditions struggling to follow prescribed regimens and often resisting unnecessary medications (18, 19). The poor adherence to pharmacological treatments has driven interest in complementary therapies, including traditional Chinese medicine (TCM) external therapies (20, 21).

In contrast to pharmacological treatments, TCM non-pharmacological and external therapies have garnered significant attention as well-tolerated alternatives. These therapies, including moxibustion, auricular therapy, acupuncture, traditional Chinese exercises (e.g., Tai Chi, Baduanjin, Qi Gong), TCM emotional care, herbal foot baths, acupoint injections, and transcutaneous acupoint electrical stimulation, are non-invasive and generally well-accepted by patients (22). While modalities like acupuncture and moxibustion rely on physical stimulation of the body’s surface, interventions such as traditional Chinese exercises and TCM emotional care are often conceptualized in modern terms as exercise or psychological interventions. However, within the theoretical framework of TCM, both physical and mind-body approaches are unified by a core therapeutic principle: regulating the flow of Qi and Blood, balancing Yin and Yang, and unblocking meridians. In the clinical management of CRF, these diverse modalities are frequently prescribed interchangeably or as parallel options within a holistic, non-pharmacological treatment strategy. Therefore, evaluating them within a single analytical framework is clinically relevant and maintains the transitivity assumption for network meta-analysis, as they are targeted at the same patient population utilizing the same underlying physiological rationale. This better adherence to TCM external therapies may be attributed to their holistic approach, lower side-effect profiles, and non-invasive nature, making them an appealing option for cancer patients.

Evidence supports several of these therapies, such as moxibustion (23), auricular acupressure (24), acupressure (25), and Tai Chi (26), for their efficacy in alleviating CRF. Currently, the diversity of external TCM treatments for CRF has led to numerous options, yet each therapy’s effectiveness varies. Existing meta-analyses mostly focus on single interventions without systematically comparing multiple therapies, and there is a scarcity of randomized controlled trials (RCTs) that directly compare these treatments. Consequently, the optimal TCM external therapy for managing CRF remains uncertain, complicating treatment decisions for healthcare professionals.

This systematic review aims to address these gaps by clarifying the comparative effectiveness of TCM external therapies for CRF. Through a network meta-analysis, we combined direct and indirect evidence to assess the efficacy of multiple TCM external therapies, ranking each based on outcome indicators (27). This approach aims to provide a clear evidence-based guide to help clinicians select the most effective TCM therapies for managing CRF.

## 2 Methods

This review adheres to the NMA guidelines for Preferred Reporting Items for Systematic Reviews and Meta-Analyses (PRISMA) (28). The study protocol is registered on the Prospero website (Registration No. CRD42023479194). There were two deviations from the registered protocol. Firstly, although the protocol specified 13 external therapies, our literature search was not restricted to these treatments and others were included. Secondly, the protocol specified that only conventional controls should be used, but trials with sham controls were also included. These deviations may have provided valuable insights into the comparative effectiveness of a broader range of TCM external therapies and the placebo effect of sham interventions, yet they also increase heterogeneity in the findings—potentially affecting the precision of effect size estimates and complicating direct comparisons between originally planned and additional therapies.

### 2.1 Search strategy

We conducted a comprehensive search across eight databases, including The Cochrane Library, PubMed, Embase, Web of Science, China National Knowledge Infrastructure (CNKI), WanFang Data, Chinese Biomedical Literature Database (CBM), and VIP’s Chinese Science and Technology Journal Database (VIP Database), for relevant literature up to April 2025. Search terms were developed in English and Chinese, incorporating MeSH terms and free-text keywords tailored to each database. Additionally, references from related studies and systematic reviews were manually reviewed for inclusion. Details of the search strategy can be found in **Supplementary Table 1**.

### 2.2 Inclusion and exclusion criteria

We included RCTs that met the following criteria: Population: Adults (aged 18 and older) diagnosed with cancer confirmed by tissue biopsy or cytology. Interventions: A range of TCM external therapies such as acupuncture, acupressure, Chinese exercises (e.g., Tai Chi, Baduanjin, Qi Gong), transcutaneous acupoint electrical stimulation, moxibustion, auricular acupressure, foot bath, TCM emotional care, acupoint injection, acupoint application, auricular press needle, warming needle, and acupoint hot ironing. Descriptions of each intervention are available in **Supplementary Table 2**. Comparisons: Usual care (standard or routine care): refers to the conventional medical treatment and practices that patients typically receive for their condition in a clinical setting. It includes standard procedures such as symptom management, medication, patient education, follow-up care, and other established protocols; waitlist, sham interventions, or different TCM external therapies from the intervention group.

Outcomes: Cancer-related fatigue (CRF) scores measured by validated scales, such as the Brief Fatigue Inventory (BFI), Functional Assessment of Chronic Illness Therapy–Fatigue (FACIT-F), Multidimensional Fatigue Symptom Inventory (MFSI), and others. Studies combining multiple TCM interventions(e.g., the studies that utilized a combination of acupuncture and acupoint application), those with incomplete data, duplicates, insufficient intervention descriptions, or non-English/Chinese publications were excluded. We limited our review to studies published in English and Chinese because these languages represent the majority of available research on TCM external therapies for cancer-related fatigue. Given the significant body of literature in these languages, particularly from China, and the authors’ proficiency in both languages, this selection ensures a thorough yet manageable scope.

### 2.3 Literature selection and data extraction

Two researchers (WXY and CSJ) independently screened and selected studies, importing records into EndNote X9.1 for duplicate removal. Titles and abstracts were reviewed based on pre-defined criteria, followed by full-text assessments for eligibility. For data extraction, a structured form was used to collect details including Country, study design, Sample size, population, Mean age, intervention specifics (type, frequency, duration), control groups, outcome measures, and funding. To ensure data accuracy and consistency, inter-reviewer agreement was evaluated using the kappa coefficient (κ). A κ value ≥ 0.75 indicated excellent agreement, 0.40-0.74 indicated moderate agreement, and < 0.40 indicated poor agreement. In this review, the kappa coefficient for data extraction between the two researchers was 0.82, reflecting excellent inter-reviewer consistency. Missing data was addressed by contacting authors at least three times. Disagreements were resolved by a third researcher (YJH).

### 2.4 Risk of bias

Risk of bias was independently assessed by two researchers (WXY and CSJ) using the revised Cochrane Risk of Bias tool for randomized trials (ROB 2.0) (21). The tool evaluates five domains of bias: bias arising from the randomization process; bias due to deviations from intended interventions; bias due to missing outcome data; bias in measurement of the outcome; and bias in selection of the reported result. Each domain was rated as ‘Low risk’, ‘Some concerns’, or ‘High risk’ based on the criteria outlined in the ROB 2.0 guidelines. The overall risk of bias for each study was determined by the highest risk level identified across the five domains. Discrepancies between reviewers were resolved through discussion to reach consensus.

### 2.5 Data analysis

Several included studies were multi-arm RCTs involving combinations of an intervention, a sham control, and a usual care group. In our network meta-analysis, multi-arm trials were handled by fully preserving the intra-study correlation of effect sizes. Importantly, “sham therapies” (e.g., sham acupuncture, sham acupressure) and “usual care” were treated as distinct, independent nodes in the network. This separation allowed us to explicitly evaluate and adjust for potential placebo effects distinct from the baseline conventional standard of care. For multi-arm trials evaluating multiple active TCM therapies simultaneously (e.g., TEAS vs. Auricular acupressure vs. control), all relevant arms were integrated into the network framework to construct both direct and indirect comparisons. Network meta-analysis was performed with Stata MP 17.0 software under a frequentist framework, utilizing standardized mean differences (SMD) and 95% confidence intervals (CI) to assess effect sizes. A network plot illustrated direct and indirect intervention comparisons. Global inconsistency was examined using inconsistency models, while node-splitting assessed local inconsistencies when closed loops were identified. When no statistical inconsistencies were present (*P* > 0.05), a consistency model was applied. Publication bias was evaluated using funnel plots, and SUCRA values ranked interventions’ effectiveness, with values near 100% indicating the highest effectiveness probability. Sensitivity analysis was conducted by excluding small sample studies (sample size ≤ 30) to assess the stability of the research results.

### 2.6 Certainty of evidence

We assessed evidence certainty with the CINeMA methodology based on the GRADE framework, covering six domains: within-study bias, reporting bias, indirectness, imprecision, heterogeneity, and incoherence (29). Each domain was rated as having no concerns, some concerns, or major concerns, with treatment effects categorized into high, moderate, low, or very low confidence levels (29).

## 3 Results

### 3.1 Study selection

**Figure 1:**
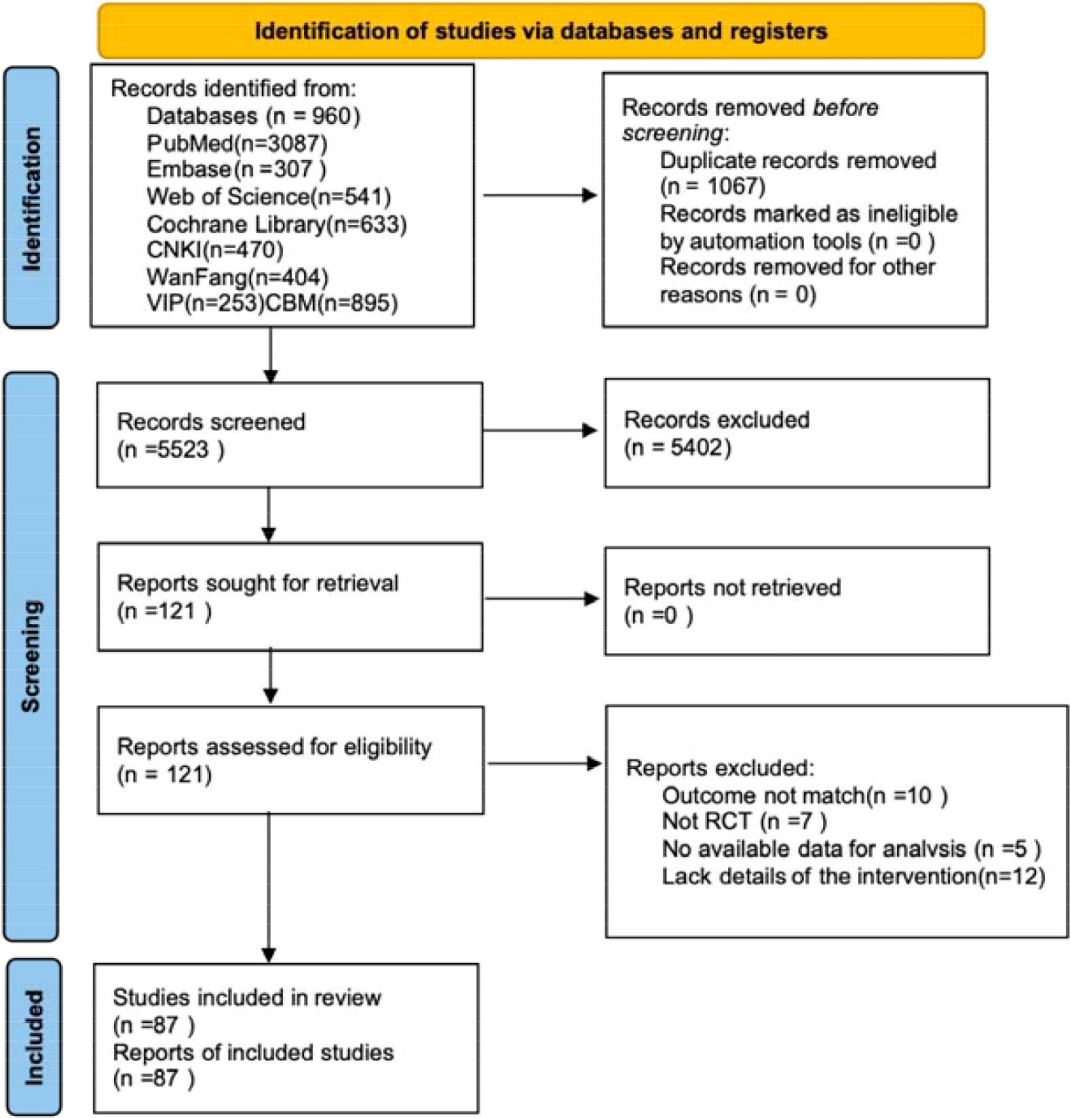
PRISMA 2020 flow diagram of study selection.

This study illustrates that the initial search yielded 6,590 records. After removing 1,067 duplicates, 5,523 were screened, with 5,402 excluded. Subsequently, the full text of the remaining 121 articles was screened, and thirty-four studies were excluded because the literatures were not RCTs, the outcome indicators did not meet the requirements of this paper, and the intervention details were insufficient. (e.g., the study did not adequately describe the nature, duration, or method of the intervention, or if there was insufficient information on the frequency, intensity, or mode of delivery).Information on studies excluded from the full-text screening process can be found in **Supplementary Table 3**. A PRISMA 2020 flow diagram (**Figure 1**) is included in this section to visualize the above screening and selection process. Ultimately, eighty-seven randomized controlled trials were included in the review (30–116).

### 3.2 Characteristics of included studies

**Supplementary Table 4** provides a detailed description of the study characteristics included in this systematic review. This review analyzed eighty-seven RCTs involving 7,028 participants across ten countries, with most studies conducted in China. The included studies covered a diverse range of malignancies. The most common classifications were mixed cancer types (n = 27), lung cancer (n = 16), breast cancer (n = 14), and gastrointestinal/colorectal cancers (n = 14). Other targeted populations included gynecological cancers (n = 5), lymphomas/leukemias (n = 3), and specific localized cancers (e.g., prostate, thyroid, oral) Interventions spanned 13 distinct TCM therapies. The specific number of studies contributing to each active intervention node is as follows: moxibustion (n = 26), acupuncture (n = 13), transcutaneous acupoint electrical stimulation (TEAS, n = 8), traditional Chinese exercises including Qi Gong (n = 7), Tai Chi (n = 4) and Baduanjin (n = 5), acupressure (n = 6), auricular acupressure (n = 5), acupoint application (n = 5), Chinese medicine foot bath (n = 3), TCM emotional care (n = 2), acupoint hot ironing (n = 1), acupoint injection (n = 1), and auricular press needle (n = 1) Commonly used measurement tools included The Brief Fatigue Inventory (BFI), Revised Piper Fatigue Scale (RPFS), Piper Fatigue Scale (PFS), The fatigue subscales of the European Organization for Research and Treatment of Cancer Quality of Life Questionnaire Core 30,EORTC QLQ-C30, and Multidimensional Fatigue Inventory (MFI).

### 3.3 Risk of bias assessment

**Figure 2:**
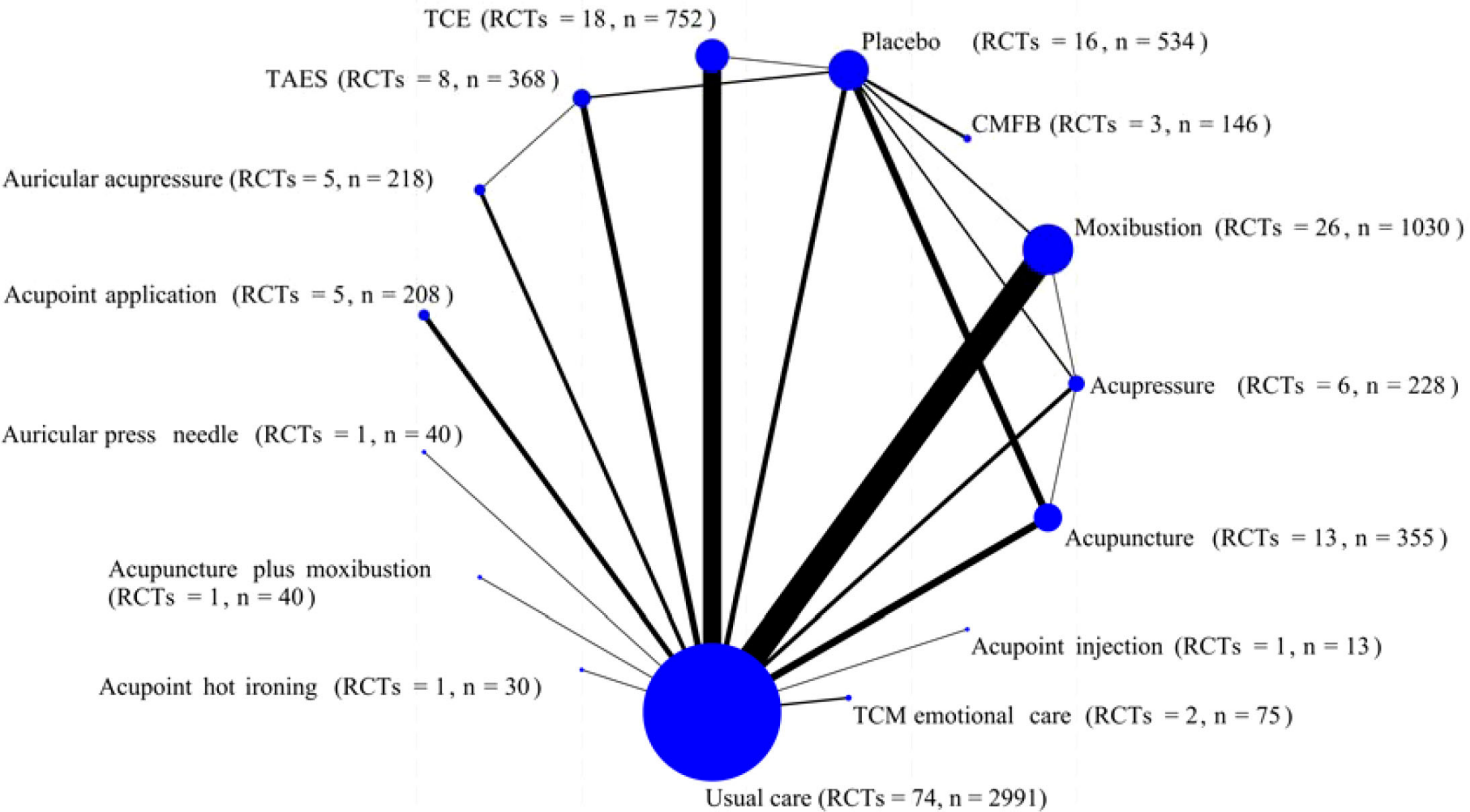
Network plot for cancer-related fatigue scores of included studies.

The assessment of 87 studies using the Cochrane ROB 2.0 tool revealed that 55.2% raised “some concerns”, primarily due to deviations from intended interventions (74.7% lacked blinding details for participants and personnel, a persistent challenge in non-pharmacological TCM trials) and missing outcome data (19.5% unexplained attrition). Overall, 28.7% were “low risk”, while 16.1% were “high risk” due to selective reporting or measurement bias. These methodological shortcomings directly undermine the internal validity of the included trials and significantly contribute to the overall low certainty of the synthesized evidence. **Figure 2**, **Supplementary Figure 1** illustrate this assessment.

### 3.4 Pairwise meta-analysis results

To robustly cross-validate the network estimates and provide a more clinically interpretable reference, we conducted standard pairwise meta-analyses for all direct comparisons available in the included studies. The results demonstrated that the majority of the investigated TCM external therapies were significantly more effective than usual care in alleviating cancer-related fatigue. In a pairwise comparison of the 13 external Chinese medical treatments, warming needle were significantly superior to acupuncture, acupressure, traditional Chinese exercises, transcutaneous acupoint electrical stimulation, and auricular acupressure. Moxibustion was significantly superior to acupuncture, traditional Chinese exercise, and transcutaneous acupoint electrical stimulation. The remaining interventions did not exhibit statistically significant distinctions. The results of the paired Meta-analyses are detailed in **Supplementary Figure 2**.

### 3.5 Network meta-analysis

#### 3.5.1 Network plot

**Figure 3:**
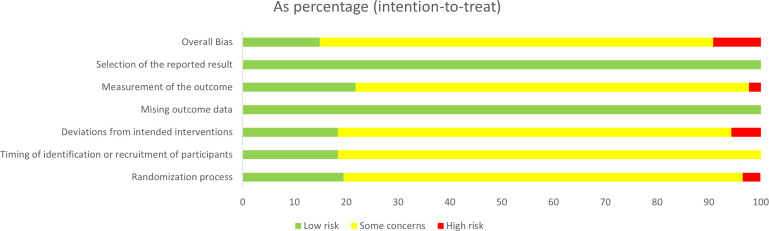
Risk of bias graph1.

A network diagram based on fifteen interventions was constructed, as shown in **Figure 3**.The diagram included fifteen nodes, representing thirteen TCM external therapies (acupuncture, acupressure, traditional Chinese exercises, transcutaneous acupoint electrical stimulation, moxibustion, auricular acupressure, Chinese medicine foot bath, traditional Chinese medicine emotional care, acupoint injection, acupoint application, auricular press needle, warming needle, acupoint hot ironing) and two comparison interventions (usual care and sham interventions). In a direct comparison of the thirteen TCM external therapies with the comparison intervention, moxibustion, traditional Chinese exercise, and acupuncture were the most frequently used intervention groups. The most frequently used comparison group was usual care, followed by sham interventions.

#### 3.5.2 Efficacy outcomes

**Figure 4:**
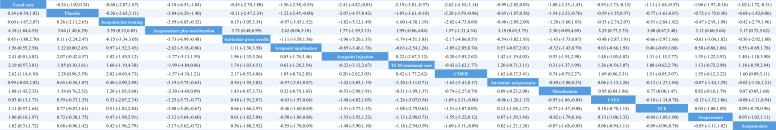
League graph for comparing traditional Chinese medicine interventions on cancer-related fatigue scores in patients with cancer.

The effectiveness of each intervention on CRF was estimated through NMA, as depicted in **Figure 4**. The results of this analysis demonstrate that acupuncture (SMD = -1.02; 95% CI: -1.72, -0.31), acupressure (SMD = -1.06; 95% CI: 1.97, -0.16), traditional Chinese exercises (SMD = -1.11; 95% CI: -1.64,- 0.57), transcutaneous acupoint electrical stimulation (SMD = -0.93; 95% CI: -1.73, -0.13), moxibustion (SMD = -1.88; 95% CI: -2.33, -1.43), Chinese medicine foot bath (SMD = -2.62; 95% CI: -4.10, -1.13), TCM emotional care (SMD = -2.19; 95% CI: -3.81, -0.57), acupressure injection (SMD = -2.41; 95% CI: -4.82, -0.01), acupoint application (SMD = -1.56; 95% CI: -2.58, -0.55), warming needle (SMD = -4.18; 95% CI: -6.53, -1.84) significantly relieved CRF when compared to usual care (*P* < 0.05). In addition, the review found that moxibustion, Chinese medicine foot bath, TCM emotional care, acupoint injection, acupoint application, warming needle were significantly more effective than the sham control group in alleviating CRF (*P* < 0.05). For clinical interpretation of the standardized mean difference (SMD) of interventions for cancer-related fatigue (CRF), we first referred to established minimal clinically important differences (MCIDs): 1.0 - 2.0 for BFI (0–10 scale), 1.0 for PFS (0–10 scale), and3.0 - 5.0 for FACIT-F (0–52 scale). There are slight differences in MCID among different studies. Here, the recognized lower limit is selected for a conservative assessment of clinical significance. We then converted SMD to raw score changes using the formula “Raw score change = |SMD| × pooled standard deviation (SD)”. Most significant SMDs (e.g., warming needle’s SMD = -4.18, Chinese medicine foot bath’s SMD = -2.62) corresponded to changes exceeding the respective MCIDs, indicating practical clinical benefits for patients.

#### 3.5.3 Rank probabilities

**Figure 5:**
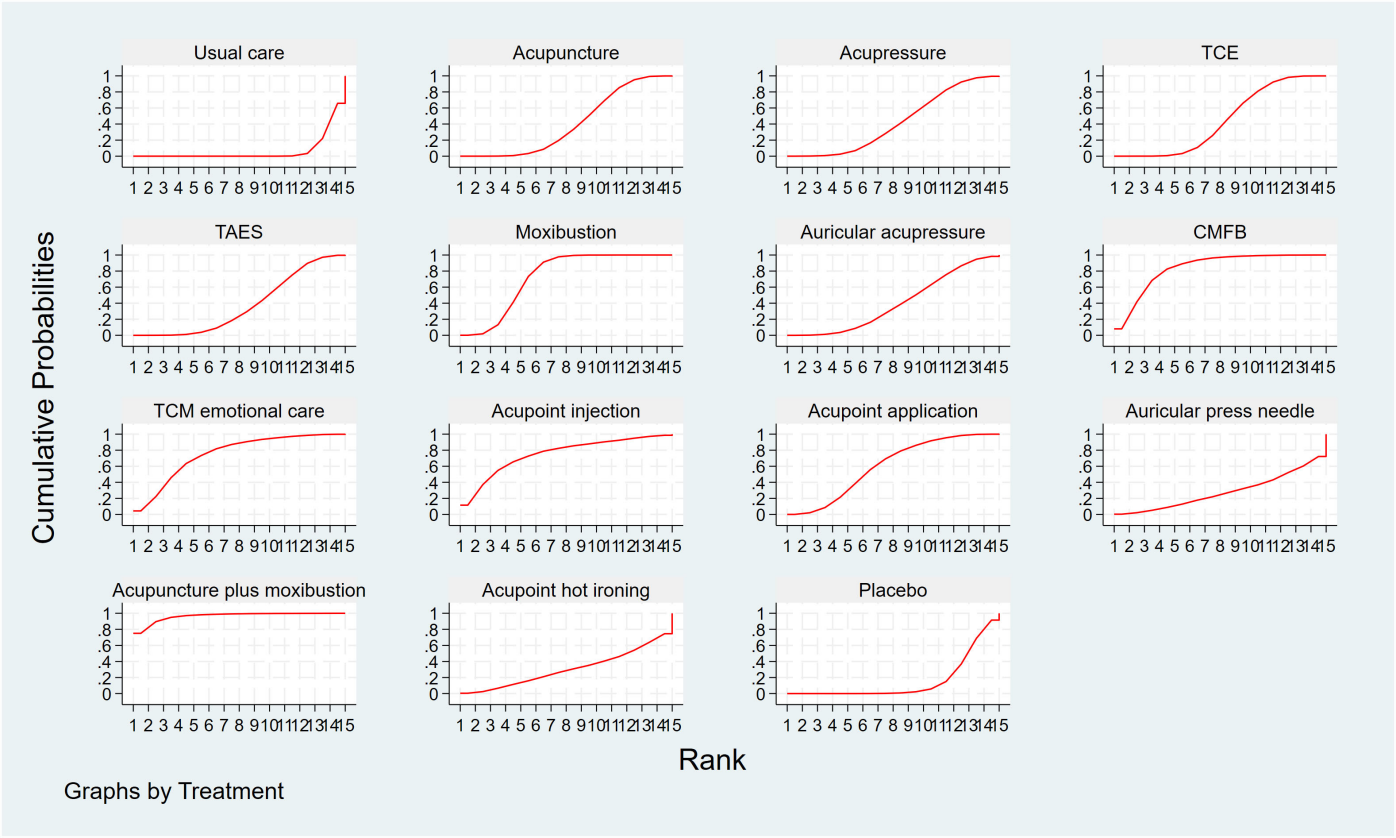
Cumulative ranking probabilities of all interventions.

The SUCRA is presented in **Figure 5**, **Table 1**. For the thirteen interventions, the SUCRA values predicted the likelihood of different interventions as optimal treatments for CRF with the following ranking of treatment effects: warming needle (96.3%), Chinese medicine foot bath (83.4%), TCM emotional care (75.1%), acupoint injections (75.1%), moxibustion (73.2%), acupoint application (60.9%), auricular acupressure (48.3%), traditional Chinese exercises (46.5%), acupressure (39.5%), acupuncture (35%), Transcutaneous acupoint electrical stimulation (34.6%), acupoint hot ironing (32.4%), auricular press needle (29.4%), usual care (10.5%), and Sham interventions (9.8%). warming needle was identified as the intervention with the highest cumulative probability for effectively alleviating CRF.

**Table 1:** Ranking probability of all interventions.

Intervening measure	Efficacy outcomes		Sensitivity analysis		Subgroup analysis (cancer types)									
					Breast cancer		Colorectal cancer		Gastric cancer		Lung cancer		Mixed cancer	
	SUCRA	Rank	SUCRA	Rank	SUCRA	Rank	SUCRA	Rank	SUCRA	Rank	SUCRA	Rank	SUCRA	Rank
Acupoint hot ironing	32.4	12	30.6	12	/	/	/	/	/	/	46.5	4	/	/
Acupoint injection	75.1	4	75.6	3	/	/	/	/	/	/	/	/	91.2	2
Acupressure	39.5	9	38	9	79.2	1	/	/	/	/	45.9	6	60.3	5
Acupuncture	35	10	32.9	10	57.1	4	/	/	/	/	97.6	1	51.1	6
CMFB	83.4	2	84	2	/	/	/	/	/	/	/	/	/	/
Moxibustion	73.2	5	73.1	5	70	2	83.8	1	100	1	45.1	7	64.8	3
Placebo	9.8	15	22.3	14	44.7	5	52.3	2	/	/	39.4	10	19.3	9
TAES	34.6	11	32.6	11	/	/	/	/	/	/	46.8	3	37.1	8
TCE	46.5	8	45.4	8	34.4	6	/	/	/	/	43.8	9	44.2	7
TCM emotional care	75.1	3	75.5	4	/	/	/	/	/	/	/	/	62.6	4
Acupoint application	60.9	6	60.8	6	63.8	3	/	/	/	/	46.3	5	95.4	1
Acupuncture plus moxibustion	96.3	1	96.9	1	/	/	/	/	/	/	/	/	/	/
Auricular acupressure	48.3	7	47.2	7	/	/	/	/	50	2	57.4	2	13	10
Auricular press needle	29.4	13	27.4	13	/	/	/	/	/	/	44.6	8	/	/
Usual care	10.5	14	7.6	15	0.8	7	13.9	3	0	3	36.5	11	11.1	11

#### 3.5.4 Sensitivity analysis

For the sensitivity analysis, small-sample studies (sample size ≤ 30) were excluded to test result stability. The findings showed that the ranking trend of intervention effectiveness was consistent with that of the full-sample analysis: warming needle (A + M) remained the optimal intervention for relieving cancer-related fatigue (CRF), while Chinese medicine foot bath and TCM emotional care still ranked high, and interventions with weaker effects such as auricular press needle and acupoint hot ironing did not have reversed rankings (**Table 1**). Indicating good stability of the full-sample network meta-analysis results in terms of sample size.

#### 3.5.5 Subgroup analysis

A subgroup analysis was conducted focusing on different cancer types, namely breast cancer, colorectal cancer, gastric cancer, lung cancer, and mixed cancers, and the results exhibited certain characteristics in terms of stability (**Table 1**). Within each cancer subgroup, although there were slight variations in the effectiveness ranking of core interventions, the overall trend remained consistent with that of the full-sample analysis. Specifically, in the breast cancer subgroup, acupressure ranked first (79.2%), followed by moxibustion (70%) and acupoint application (63.8%); in the colorectal cancer subgroup, moxibustion took the top spot (83.8%); in the gastric cancer subgroup, moxibustion also ranked first (100%), followed by auricular acupressure (50%); in the lung cancer subgroup, acupuncture was the most effective (97.6%), followed by auricular acupressure (57.4%) and transcutaneous acupoint electrical stimulation (46.8%); and in the mixed cancer subgroup, acupoint application ranked first (95.4%), followed by acupoint injection (91.2%) and moxibustion (64.8%). Notably, usual care consistently ranked at the bottom of the effectiveness scale across all subgroups. Additionally, due to the limited number of included studies or small sample sizes in some subgroups, the estimation accuracy of the effect sizes of certain interventions was relatively low. However, the inconsistency test within each subgroup revealed no significant contradictions and there were no cases where the conclusions completely contradicted those of the full-sample analysis, further confirming the partial stability of the overall study results across different cancer subgroups. Nevertheless, the stability of results for subgroups with small sample sizes or a limited number of interventions still requires verification through more high-quality studies. Nevertheless, it must be strongly emphasized that due to the limited number of included studies and the small sample sizes within these specific strata, the estimation accuracy is compromised. Therefore, the conclusions drawn from these small-sample subgroups are strictly exploratory and should be utilized for reference only, pending verification through more robust, high-quality RCTs.

### 3.6 Publication bias

Publication bias was assessed in the eighty-seven included studies using a funnel plot [(**Supplementary Figures 3A, B)**]. Funnel plots revealed asymmetry, suggesting potential publication bias (Egger test, *P* < 0.05). This potential publication bias may have a multifaceted impact on the validity of the study findings. On one hand, it could lead to an overestimation of the effectiveness of certain TCM external therapies for CRF. Studies with statistically significant positive results are more likely to be submitted and published, while those with non-significant or negative findings may remain unpublished, creating a skewed dataset. For example, interventions like warming needle, which was ranked as the most effective, might have had more published positive studies, exaggerating its true efficacy. On the other hand, this bias could undermine the reliability of the comparative rankings of different therapies. The relative effectiveness of less frequently studied interventions, such as auricular press needle or acupoint hot ironing, might be inaccurately represented due to the lack of unpublished data that could provide a more balanced perspective. Additionally, the overall consistency of the network meta-analysis results, despite passing the inconsistency test, could be compromised by publication bias, as the excluded studies might have introduced additional variability or corrected the observed effect sizes. This makes it crucial for readers to interpret the study’s conclusions with caution, especially when considering the clinical application of the top-ranked therapies.

### 3.7 Inconsistency test

The consistency of the included studies was evaluated on a global and local level. The results of the global inconsistency test indicated that there were no significant inconsistencies (χ2 = 4.63, *P* = 0.990). A node split test was performed to analyze local inconsistency, revealing no significant disparities between the direct and indirect effects of various TCM external therapies (*P* > 0.05). Consequently, a consistency model was employed in the NMA. Forest plots for the network were generated based on effect size [refer to **Supplementary Figure 3C**)].

### 3.8 Evidence quality assessment

Evidence quality in this review was assessed using the CINeMA system. Despite the large number of studies in the network, confidence in the effect is low. This is largely due to the high within-study bias described in the risk of bias summary chart. Many of the studies focused on only one cancer type, older adults, and inconsistent measurement tools for outcome indicators, so most study groups had some concerns about indirect comparisons. Overall, warming needle, and Chinese medicine foot bath demonstrated low certainty compared to usual care. Acupuncture, Chinese medicine foot bath and warming needle showed low certainty compared to sham interventions. Evidence quality for other interventions was rated as very low. For detailed assessment results, refer to **Supplementary Tables 5**, **6**.

## 4 Discussion

### 4.1 Overview of network meta-analysis results

To our knowledge, this review represents the most comprehensive network meta-analysis comparing the effectiveness of thirteen TCM external therapies for CRF, synthesizing data from eighty-seven randomized controlled trials and 7,028 participants. The results of the inconsistency test showed no significant inconsistency either overall or locally, indicating the stability of the review findings. As established primarily by our direct pairwise meta-analyses (Section 3.3), direct evidence supports the preliminary efficacy of several modalities over standard care. Overall, various TCM methods, including acupuncture, acupressure, traditional Chinese exercises, transcutaneous acupoint electrical stimulation, moxibustion, Chinese medicine foot baths, TCM emotional care, acupoint injections, and warming needle, showed benefits over standard care or waitlist control. Building upon this direct evidence, our network model generated a probabilistic hierarchy. Based on the SUCRA values, the top-ranked interventions were warming needle, Chinese medicine foot baths, TCM emotional care, acupoint injections, and moxibustion. However, it is crucial to emphasize that the certainty of evidence for these top-ranked interventions, particularly warming needle, is rated as low to very low. Furthermore, as noted in our results, the unusually large effect sizes (e.g., SMD = -4.18) generated by the models likely reflect the inherent heterogeneity of subjective fatigue scales, variations in baseline severity, and potential small-study effects, rather than representing a strictly proportional and realistic clinical magnitude. Coupled with the identified publication bias, these factors limit the robustness of these conclusions. Therefore, our current findings and the resulting treatment rankings are strictly exploratory and hypothesis-generating. They should not be interpreted as definitive treatment rankings or as robust clinical guidelines. Any subsequent interpretations of potential therapeutic mechanisms should also be viewed cautiously through this exploratory lens.

### 4.2 Acupuncture combined with moxibustion: the highest-ranked intervention

In the results of the analysis of ranking probabilities, warming needle had the highest ranking probability, suggesting that it could potentially be a highly effective external TCM treatment for patients with CRF. A previous study by Du et al. also noted that warming needle has the potential to reduce the severity of CRF and improve the quality of life of colorectal cancer patients, which is consistent with the results of this review (117). The findings of the network meta-analysis indicated that the combination of acupuncture and moxibustion was more effective than acupuncture alone, which is consistent with the results reported by Li et al (118).

To facilitate clinical application, a brief summary of the commonly applied protocols from the included studies is warranted. Typically, warming needle therapy for CRF involves sessions lasting 20 to 30 minutes, administered 2 to 3 times per week over a course of 2 to 4 weeks. Clinicians frequently select core acupoints associated with tonifying Qi and Blood, such as Zusanli (ST36), Guanyuan (CV4), Qihai (CV6), and Sanyinjiao (SP6) (39). Regarding potential mechanisms, current literature provides hypotheses rather than definitive conclusions. Some researchers propose that Acupuncture stimulation has been demonstrated to might promote blood circulation, regulate serum inflammatory factors, modulate the neuroendocrine system, and maintain body homeostasis. These effects have been shown to alleviate symptoms of fatigue (119–121). Moxibustion produces thermal stimulation to the corresponding parts of the body through heat and light radiation, which improves local blood circulation, promotes blood and Qi flow throughout the entire body, and improves metabolism (122). Furthermore, moxibustion can enhance the body’s immunity and its ability to resist disease (123). When using acupuncture in combination with moxibustion, the warmth generated by burning moxa can be conducted through the needles into the body, providing warmth to the corresponding acupoints, enhancing the circulation of qi and blood, and effectively alleviating symptoms of fatigue (124, 125). For instance, a single interventional study involving patients undergoing colorectal cancer treatment suggested that the combined approach of acupuncture and moxibustion might offer potential synergistic benefits compared to single therapies (126). Despite these promising theoretical frameworks and initial ranking probabilities, it is imperative to reiterate that the certainty of evidence for warming needle is low. This stems from issues such as within-study bias, inconsistent measurement tools, and limited direct comparisons—means that its superiority cannot be definitively established. Overreliance on this intervention without further validation may lead to suboptimal clinical decisions. Although the results of this review indicate that warming needle may be the most efficacious treatment for CRF, the certainty of the evidence was low, and the comparisons were indirect. However, our findings should be interpreted cautiously due to the low certainty of evidence and limited direct comparisons in the included studies. Further trials are needed to confirm these results.

### 4.3 Chinese medicine foot bath: a promising treatment

In addition, our findings suggest that Chinese medicine foot bath have great potential, which ranked second in effectiveness. Xu et al. demonstrated that Qi-benefiting Chinese medicine foot bath significantly alleviate CRF and improve the quality of life of cancer patients, consistent with our findings (127). The reason for this may be that the soles of the feet have more than three hundred acupoints and sixty-seven reflex zones, which reflect the human body’s health (128). Chinese medicine foot bath utilize transdermal drug delivery, allowing the herbs to be absorbed through the skin (129). Additionally, the warmth of the herbal solution can dilate the capillaries in the feet, promoting the circulation of Qi and blood, enhancing drug absorption, improving overall blood circulation, regulating yin-yang balance, and strengthening the body’s immune system (43, 79).

Based on the studies reviewed, a standard clinical protocol for Chinese medicine foot baths typically involves immersing the patients’ feet and lower legs in an herbal decoction (approximately 1000 mL) maintained at a monitored temperature of 40 °C to 50 °C. Treatment sessions generally last 20 to 30 minutes and are conducted once daily for a duration of 1 to 2 weeks (41, 76, 77). In this review, we observed that the commonly used herbs in traditional Chinese medicine foot bath for treating CRF include Astragalus membranaceus, Rhizoma Chuanxiong, Caulis spatholobi, Liquorice, Peony, etc. Traditional Chinese medicine foot bath are a relatively simple and non-invasive form of treatment, which makes them an ideal option for patients who are being treated at home (130). Yet, similar to warming needle, the evidence certainty for Chinese medicine foot bath is low. Variability in herb combinations, bath temperature, and duration across included studies further complicates the generalization of its efficacy, and more standardized, high-quality trials are needed to confirm its consistent benefits. However, it is important to note that the temperature and duration of the foot baths should be carefully monitored to ensure optimal results.

### 4.4 TCM emotional care: the impact of emotional well-being on CRF

TCM emotional care ranked third, underscoring the impact of emotional well-being on CRF. The advent of cancer and the subsequent lifelong treatment regimen impose a significant medical burden on patients, leading to alterations in mood and roles for some patients, which further exacerbates the psychological burden and results in elevated CRF (131, 132).TCM emotional care focuses on negative emotions (e.g., anxiety, overthinking, sadness, depression, etc.) commonly experienced by CRF patients (133).

In clinical practice, as observed in our included trials, TCM emotional care protocols are highly individualized. The standard approach typically involves establishing dedicated communication sessions integrated into routine care. Core techniques include clarifying doubts to reduce disease-related anxiety, applying the principle of “overcoming emotions with emotions” (e.g., using joy to overcome sadness), emotional catharsis, and employing Five-Element music therapy (often prescribed for 15 to 30 minutes daily) to induce relaxation (46, 61).The dialectical use of the various measures of TCM emotional care, including clarifying doubts and puzzles, transferring emotions, overcoming emotions with emotions, catharsis and relief of depression, using five element music therapy, can alleviate the fatigue associated with these negative emotions (134). While this intervention aligns with a holistic approach to CRF management, its evidence certainty is also limited. The subjective nature of emotional and fatigue assessments, along with potential variability in how TCM emotional care is delivered (e.g., frequency of sessions, therapist training), introduces uncertainty about its true effectiveness, highlighting the need for more rigorous study designs.

### 4.5 Cautious optimism regarding less-represented interventions

However, we must approach the less-represented interventions, such as auricular acupressure and acupoint application, with cautious optimism. While they demonstrate promise, the smaller sample sizes in these comparisons may influence effect estimates, signaling a need for further trials to strengthen confidence in these findings. This caution extends to all interventions in the review, as even the top-ranked ones suffer from low evidence certainty. For less-represented therapies, the combination of small sample sizes and potential publication bias (evidenced by funnel plot asymmetry) makes their preliminary positive results even more tentative, and they should not be prioritized in clinical practice without additional research.

### 4.6 External therapies and key acupoint applications

In TCM, external therapies like acupuncture, acupressure, moxibustion, transcutaneous electrical stimulation, and acupoint hot ironing work by stimulating specific acupoints and meridians to relieve CRF. In our previous statistics, we found that the main acupoints commonly used to treat fatigue symptoms in cancer patients are Zusanli (ST36), Sanyinjiao (SP6), Taixi (KI3), Hegu (LI4), Qihai (CV6), Guanyuan (CV4), Zhongwan (RN12), and Pishu (BL20), which are similar to the acupoints chosen from the previous reviews, these acupoints can provide useful information for clinical operation (135). Zusanli (ST36) is an acupoint of the Stomach Meridian of Foot Yangming, with the effects of replenishing qi and blood, harmonizing yuan qi, and improving deficiency. Guanyuan (CV4) is the collection point of the Small Intestine Meridian, located on the Ren Meridian, which can stabilize the foundation, nourish the kidneys, replenish essence and blood, and warm Yang. Sanyinjiao (SP6), a point on the Spleen Meridian of Foot Taiyin, regulates the functions of the kidneys, liver, spleen, and other organs, and connects the heart and kidneys. Qihai (CV6) is the sea of yuan qi, which can replenish and regulate qi. CRF can be effectively relieved by stimulating the above acupoints. Despite the consistency in acupoint selection across studies, the low evidence certainty for the therapies using these acupoints means that the specific mechanism linking acupoint stimulation to CRF relief remains incompletely validated. Future research should not only confirm efficacy but also explore the biological pathways underlying these effects to strengthen the evidence base.

### 4.7 Proposed modern biological mechanisms of TCM therapies for CRF

Traditional TCM theory explains external therapies through regulation of Qi and blood; framing these interventions within the modern pathophysiology of cancer-related fatigue (CRF) offers a more testable biomedical rationale (136, 137). CRF is multifactorial and is commonly linked to systemic inflammation, neuroendocrine/autonomic dysregulation, and (emerging evidence suggests) gut–brain axis alterations (136, 137). TCM-related external or nonpharmacologic modalities (e.g., acupuncture/TEAS, auricular stimulation, and mind–body exercise such as Tai Chi) may relieve fatigue by intersecting with these pathways (136). Specifically, inflammatory biomarkers (e.g., IL-6, TNF-α, CRP) correlate with fatigue severity (138), and vagus-nerve–mediated anti-inflammatory signaling (the “inflammatory reflex”) provides a plausible mechanism for acupoint-based neuromodulation (139, 140). In addition, CRF has been associated with HPA-axis and autonomic imbalance; altered diurnal cortisol rhythms have been reported in fatigued cancer survivors (141), and mind–body practices may improve autonomic/stress-related physiology while modulating inflammatory biology (142). Finally, altered gut microbiota has been proposed as a contributor to fatigue-related symptoms via immune and neuroactive signaling, supporting the plausibility of gut–brain axis involvement (145). These mechanistic links remain largely inferential and should be validated by larger translational studies integrating biomarker panels and multi-omics approaches (136, 137).

## 5 Limitations

Several limitations should be considered in interpreting the results of this review. Firstly, The deviations from the registered protocol, including the inclusion of additional treatments beyond the 13 specified and the use of sham control groups, may have introduced some variability in the results. While the broader treatment inclusion may enhance generalizability, it could also increase heterogeneity, potentially affecting the precision of the findings. The use of sham controls, though not originally planned, may have led to more conservative estimates of treatment efficacy by accounting for sham interventions effects, and by more effectively explaining the sham interventions effect, it could also improve the internal validity of the study results. Despite these deviations, we believe the overall conclusions remain valid, though adherence to the protocol in future studies is recommended to minimize potential bias. Secondly, the clinical heterogeneity between studies, including variations in interventions, duration, cancer types, and measurement tools, cannot be ignored. However, limited by the number of studies, further subgroup analyses of these variables were not conducted. Subgroup analysis based on different variables can be conducted in future effectiveness evaluations. If there is enough research published in the future, we will also update and conduct subgroup analysis. Furthermore, the credibility of the findings is contingent on the quality of the included literature. Some studies included in this review did not explicitly provide information on randomization, allocation concealment, or reasons for missing data, which could potentially reduce the reliability of the findings. Despite our best efforts to search for all relevant trials, we were unable to guarantee that all trials investigating external Chinese medicine treatments for CRF were included. Finally, the limited number of studies and relatively small sample sizes may have resulted in an overestimation of intervention effects. These limitations directly contribute to the low evidence certainty observed across most interventions, particularly top-ranked ones like warming needle. They highlight that the current findings are preliminary and that the true effectiveness of TCM external therapies for CRF may be less pronounced than suggested by this review.

## 6 Implications for clinical practice and research

### 6.1 Advice for healthcare professionals

TCM external therapies like acupressure, auricular acupressure, Chinese exercises, and foot baths can help manage CRF. While these interventions are generally well-tolerated and can often be administered at home, their clinical application must be guided by the current evidence base. These treatments are simple, cost-effective, and safer than medications in terms of dependence. Healthcare professionals should offer adequate training to patients and their families on how to apply these therapies correctly at home. Regular follow-ups are important to track effectiveness.

For invasive or clinically administered therapies (e.g., acupuncture), aseptic techniques must be followed, and the skin should be checked before treatment. Most importantly, given the low evidence and the exploratory nature of our findings, healthcare professionals should avoid positioning any single TCM external therapy as a definitive or standalone treatment. Instead, they should frame these therapies as adjunctive, individualized choices, and prioritize sharing the uncertainty of evidence with patients to support informed and shared decision-making.

### 6.2 Advice for patients

Patients can use TCM therapies like acupressure, auricular acupressure, and foot baths to reduce CRF. These are easy to learn and can be practiced at home. Patients should receive training from healthcare providers to ensure they apply the therapies correctly, and family members can help. It is crucial to recognize operational risks and contraindicated populations. For example, when applying thermal therapies such as foot baths or moxibustion, patients, particularly those with chemotherapy-induced peripheral neuropathy and potentially diminished temperature sensitivity, should closely monitor the temperature of the water and moxa to reduce the risk of inadvertent burns or scalds. In addition, vigorous acupressure or localized thermal interventions are contraindicated in individuals with active skin infections, open wounds, severe thrombocytopenia or bleeding tendencies, and severe deep vein thrombosis. Regular check-ins with doctors are recommended to assess progress.

Some therapies, like acupuncture, require professional administration. Patients should follow healthcare advice to ensure safety and effectiveness.

### 6.3 Advice for researchers

More high-quality, large-scale studies are needed to directly compare the effectiveness of different TCM external therapies for CRF. Crucially, future research must systematically address the current deficit in safety data. Researchers should implement standardized safety reporting protocols to actively monitor and document adverse reactions, such as skin scalds, bleeding, localized infections, or allergic reactions to herbal applications. Comprehensive evaluations of patient tolerance, drop-out rates due to adverse events, and specific contraindications should be robustly reported to build a reliable risk-benefit profile. Future research should explore how these therapies work, their long-term effects, and how they can be incorporated into regular cancer treatment plans. Safety, patient acceptance, and clinical benefits should also be investigated to strengthen the evidence base. A critical priority for future research is addressing the limitations that undermine evidence certainty(such as improving randomization methods, standardizing intervention protocols).

## 7 Conclusion

The findings of this review indicate that TCM external therapy can effectively alleviate CRF. Among all TCM external therapies included warming needle may be the most effective treatment for CRF. But, the low certainty of evidence (assessed via CINeMA) must be emphasized—this low confidence means the results should be interpreted with great caution. The findings of this review need further validation through larger and more rigorous clinical trials. These findings can improve medical professional’ clinical decision-making and offer an evidence-based foundation for selecting optimal interventions in clinical practice.

## Data Availability

The original contributions presented in the study are included in the article/**Supplementary Material**. Further inquiries can be directed to the corresponding author.

## Supplementary material

Supplementary Figure 1 The risk of bias for each study.

Supplementary Figure 2 Pairwise meta-analysis results. A, Usual care; B, Acupuncture; C, Acupressure; D, Traditional Chinese exercises; E, Transcutaneous acupoint electrical stimulation; F, Moxibustion; G, Auricular acupressure; H, Chinese medicine foot bath; I, TCM emotional care; J, Acupoint injection; K, Acupoint application; L, Auricular press needle; M, Acupuncture plus Moxibustion; N, Acupoint hot ironing; O, Sham intervention.

Supplementary Figure 3 Network funnel and Network forest plots. **(A)** Network funnel plot of included studies. A, Usual care; B, Acupuncture; C, Acupressure; D, Traditional Chinese exercises; E, Transcutaneous acupoint electrical stimulation; F, Moxibustion; G, Auricular acupressure; H, Chinese medicine foot bath; I, TCM emotional care; J, Acupoint injection; K, Acupoint application; L, Auricular press needle; M, Warming needle; N, Acupoint hot ironing; O, sham interventions. **(B)** Egger’s test. **(C)** Network forest plots of included studies. Abbreviations: 1, Usual care; 2, Acupuncture; 3, Acupressure; 4, Traditional Chinese exercises; 5, Transcutaneous acupoint electrical stimulation; 6, Moxibustion; 7, Auricular acupressure; 8, Chinese medicine foot bath; 9, TCM emotional care; 10, Acupoint injection; 11, Acupoint application; 12, Auricular press needle; 13, Warming needle; 14, Acupoint hot ironing; 15, sham interventions.

Supplementary File 1 PRISMA_2020_checklist.

Supplementary File 2 Model Parameters.

Supplementary File 3 the inconsistency testing.
