# Supplementary figures and images for: Efficacy of traditional Chinese medicine external therapy on cancer-related fatigue: a systematic review and network meta-analysis
Source: Front Oncol. 2026 Apr 22;16:1806355. doi: 10.3389/fonc.2026.1806355 (PMC13143725; doi:10.3389/fonc.2026.1806355)

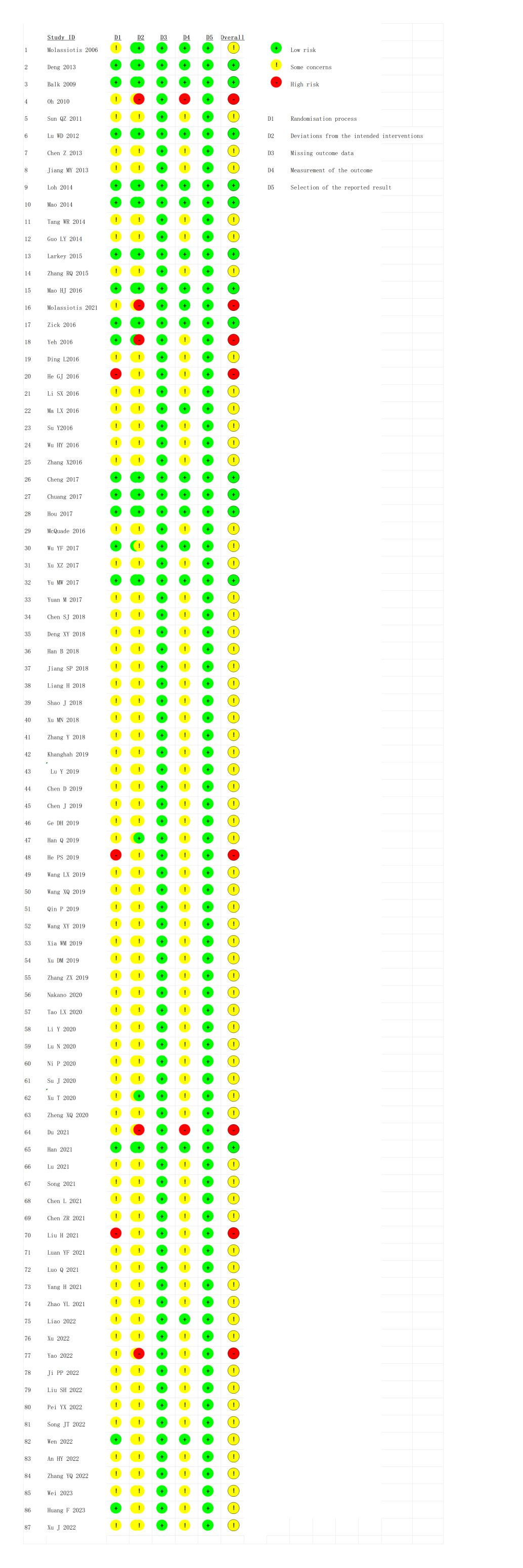

Supplement: Supplementary Figure 1 — The risk of bias for each study. [file Image1.tif]

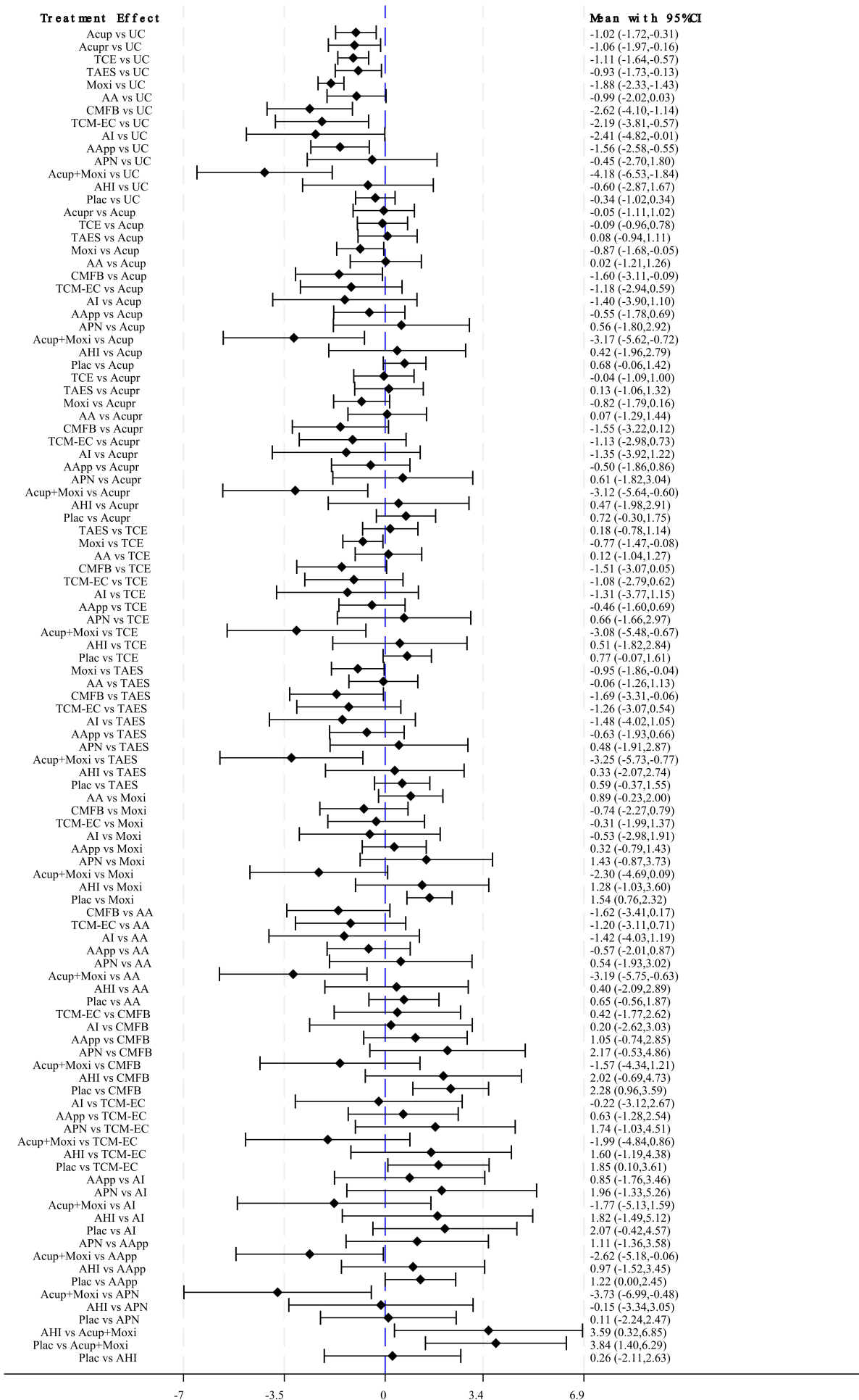

Supplement: Supplementary Figure 2 — Pairwise meta-analysis results. A, Usual care; B, Acupuncture; C, Acupressure; D, Traditional Chinese exercises; E, Transcutaneous acupoint electrical stimulation; F, Moxibustion; G, Auricular acupressure; H, Chinese medicine foot bath; I, TCM emotional care; J, Acupoint injection; K, Acupoint application; L, Auricular press needle; M, Acupuncture plus Moxibustion; N, Acupoint hot ironing; O, Sham intervention. [file Image2.pdf]

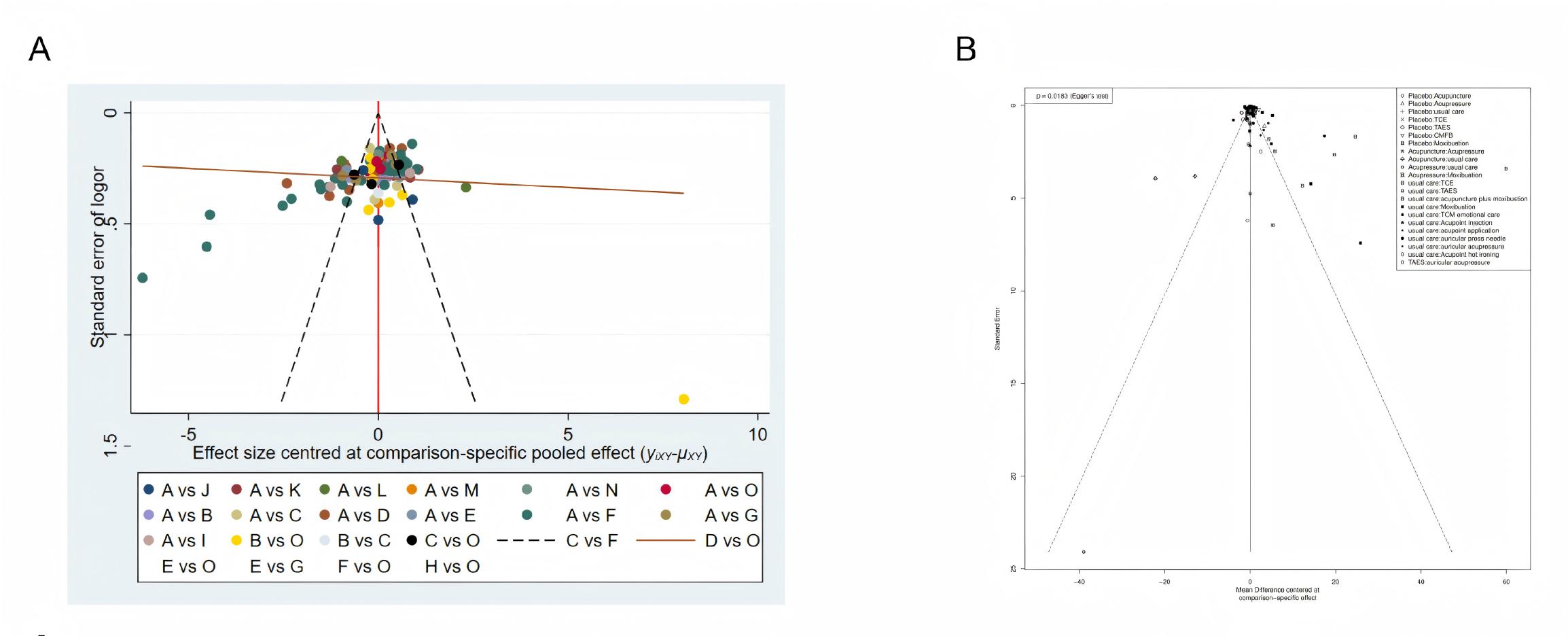

Supplement: Supplementary Figure 3 — Network funnel and Network forest plots. (A) Network funnel plot of included studies. A, Usual care; B, Acupuncture; C, Acupressure; D, Traditional Chinese exercises; E, Transcutaneous acupoint electrical stimulation; F, Moxibustion; G, Auricular acupressure; H, Chinese medicine foot bath; I, TCM emotional care; J, Acupoint injection; K, Acupoint application; L, Auricular press needle; M, Warming needle; N, Acupoint hot ironing; O, sham interventions. (B) Egger’s test. (C) Network forest plots of included studies. Abbreviations: 1, Usual care; 2, Acupuncture; 3, Acupressure; 4, Traditional Chinese exercises; 5, Transcutaneous acupoint electrical stimulation; 6, Moxibustion; 7, Auricular acupressure; 8, Chinese medicine foot bath; 9, TCM emotional care; 10, Acupoint injection; 11, Acupoint application; 12, Auricular press needle; 13, Warming needle; 14, Acupoint hot ironing; 15, sham interventions. [file Image3.jpg]
