# Supplementary material for: Efficacy of traditional Chinese medicine external therapy on cancer-related fatigue: a systematic review and network meta-analysis
Source: Front Oncol. 2026 Apr 22;16:1806355. doi: 10.3389/fonc.2026.1806355 (PMC13143725; doi:10.3389/fonc.2026.1806355)
Supplement: Supplementary file 7 [file Table1.docx]

**Supplementary table 1** The search strategy of database.

| **Database** | **Search Strategies** | **Results** |
| --- | --- | --- |
| **PubMed** | \| #1 \| "Neoplasms"[Mesh] OR "Carcinoma"[Mesh] \| \| --- \| --- \| \| #2 \| (((((((((Cancer[Title/Abstract]) OR (tumor[Title/Abstract])) OR (carcinoma[Title/Abstract])) OR (oncology[Title/Abstract])) OR (neoplasm[Title/Abstract])) OR (neoplasia*[Title/Abstract])) OR (malignanc*[Title/Abstract])) OR (malignant neoplasm[Title/Abstract])) OR (neoplasm*,malignant[Title/Abstract])) \| \| #3 \| #1 OR #2 \| \| #4 \| (((((((((((("Medicine, Chinese Traditional"[Mesh]) OR "Acupuncture"[Mesh])) OR "Electroacupuncture"[Mesh]) OR "Moxibustion"[Mesh]) OR "Transcutaneous Electric Nerve Stimulation"[Mesh]) OR "Acupuncture, Ear"[Mesh]) OR "Auriculotherapy"[Mesh]) OR "Cupping Therapy"[Mesh]) OR "Qigong"[Mesh]) OR "Tai Ji"[Mesh])) OR "Complementary Therapies"[Mesh] \| \| #5 \| ((((((((((((((((((((((((((((((((((((((((Medicine, Chinese Traditional[Title/Abstract]) OR (Traditional Chinese Medicine[Title/Abstract])) OR (TCM[Title/Abstract])) OR (Chinese External Medicine[Title/Abstract])) OR (Acupuncture[Title/Abstract])) OR (electric-acupuncture[Title/Abstract])) OR (Electro-acupuncture[Title/Abstract])) OR (Electroacupuncture[Title/Abstract])) OR (needling[Title/Abstract])) OR (moxibustion[Title/Abstract])) OR (Auricular therapy[Title/Abstract])) OR (acupoint massage[Title/Abstract])) OR (acupoint injection[Title/Abstract])) OR (point application therapy[Title/Abstract])) OR (catgut embedding therapy[Title/Abstract])) OR (acupoint embedding[Title/Abstract])) OR (transcutaneous electric nerve stimulation[Title/Abstract])) OR (auricular acupuncture[Title/Abstract])) OR (ear acupoint[Title/Abstract])) OR (ear point bean-pressing[Title/Abstract])) OR (auriculotherapy[Title/Abstract])) OR (Tuina[Title/Abstract])) OR (cupping[Title/Abstract])) OR (foot bath[Title/Abstract])) OR (Fuming Therapy[Title/Abstract])) OR (fumigation[Title/Abstract])) OR (Traditional Chinese medicine emotional care[Title/Abstract])) OR (TCM emotional therapy[Title/Abstract])) OR (five elements music therapy[Title/Abstract])) OR (Qigong therapy[Title/Abstract])) OR (qigong[Title/Abstract])) OR (taijiquan[Title/Abstract])) OR (taichi[Title/Abstract])) OR (shadow boxing[Title/Abstract])) OR (baduanjin[Title/Abstract])) OR (Yijinjing[Title/Abstract])) OR (Traditional Chinese Exercises[Title/Abstract])) OR (TCM exercise therapies[Title/Abstract])) OR (complementary therapies[Title/Abstract])) OR (Traditional Chinese medicine sticking[Title/Abstract])) \| \| #6 \| #4 OR #5 \| \| #7 \| "Fatigue"[Mesh] \| \| #8 \| ((((((((((((((Fatigue[Title/Abstract]) OR (lassitude[Title/Abstract])) OR (cancer fatigue[Title/Abstract])) OR (cancer related fatigue[Title/Abstract])) OR (Cancer-related fatigue[Title/Abstract])) OR (cancer-induced fatigue[Title/Abstract])) OR (Chemotherapy-related fatigue[Title/Abstract])) OR (Radiotherapy-related fatigue[Title/Abstract])) OR (fatigue after chemotherapy[Title/Abstract])) OR (tumor associated fatigue[Title/Abstract])) OR (CRF[Title/Abstract])) OR (treatment-related fatigue[Title/Abstract])) OR (tired*[Title/Abstract])) OR (asthenia[Title/Abstract])) \| \| #9 \| #7 OR #8 \| \| #10 \| "Controlled Clinical Trials as Topic"[Mesh] \| \| #11 \| (((("Controlled Clinical Trial" [Publication Type]) OR ("Randomized Controlled Trial" [Publication Type])) OR (randomized[Title/Abstract])) OR (placebo[Title/Abstract])) OR (randomly[Title/Abstract])) OR (trial[Title])) \| \| #12 \| #10 OR #11 \| \| #13 \| **#3** AND **#6** AND **# 9** AND **#12** \| | 3087 |
| **Embase** | \| #1 \| 'malignant neoplasm'/exp' OR 'neoplasm'/exp OR 'carcinoma'/exp' OR oncology'/exp \| \| --- \| --- \| \| #2 \| 'cancer':ab,ti OR 'tumor':ab,ti OR 'carcinoma':ab,ti OR 'oncology':ab,ti OR 'neoplasm':ab,ti OR 'neoplasia*':ab,ti OR 'malignanc':ab,ti OR 'malignant neoplasm':ab,ti OR 'neoplasm*,malignant':ab,ti \| \| #3 \| #1 OR #2 \| \| #4 \| 'chinese medicine'/exp OR 'acupuncture'/exp OR 'electroacupuncture'/exp OR 'moxibustion'/exp OR 'transcutaneous electrical nerve stimulation'/exp OR 'auricular acupuncture'/exp OR 'cupping therapy'/exp OR 'foot bath'/exp OR 'qigong'/exp OR 'tai chi'/exp OR 'alternative medicine'/exp \| \| #5 \| 'medicine, chinese traditional':ab,ti OR 'traditional chinese medicine':ab,ti OR 'TCM':ab,ti OR 'chinese external medicine':ab,ti OR 'acupuncture':ab,ti OR 'Electro-acupuncture':ab,ti OR 'electric-acupuncture':ab,ti OR 'electroacupuncture':ab,ti OR 'needling':ab,ti OR 'moxibustion':ab,ti OR 'auricular therapy':ab,ti OR 'acupoint massage':ab,ti OR 'acupoint injection':ab,ti OR 'point application therapy':ab,ti OR 'catgut embedding therapy':ab,ti OR 'acupoint embedding':ab,ti OR 'transcutaneous electric nerve stimulation':ab,ti OR 'auricular acupuncture':ab,ti OR 'ear acupoint':ab,ti OR 'ear point bean-pressing':ab,ti OR 'auriculotherapy':ab,ti OR 'tuina':ab,ti OR 'cupping':ab,ti OR 'foot bath':ab,ti OR 'fuming therapy':ab,ti OR 'fumigation':ab,ti OR 'traditional chinese medicine emotional care':ab,ti OR 'tcm emotional therapy':ab,ti OR 'five elements music therapy':ab,ti OR 'qigong therapy':ab,ti OR 'qigong':ab,ti OR 'taijiquan':ab,ti OR 'taichi':ab,ti OR 'shadow boxing':ab,ti OR 'baduanjin':ab,ti OR 'yijinjing':ab,ti OR 'traditional chinese exercises':ab,ti OR 'TCM exercise therapies':ab,ti OR 'complementary therapies':ab,ti OR 'traditional chinese medicine sticking':ab,ti \| \| #6 \| #4 OR #5 \| \| #7 \| 'fatigue'/exp OR 'cancer fatigue'/exp OR 'cancer related fatigue'/exp \| \| #8 \| 'fatigue':ab,ti OR 'lassitude':ab,ti OR 'cancer fatigue':ab,ti OR 'cancer related fatigue':ab,ti OR 'cancer-related fatigue':ab,ti OR 'cancer-induced fatigue':ab,ti OR 'chemotherapy-related fatigue':ab,ti OR 'radiotherapy-related fatigue':ab,ti OR 'fatigue after chemotherapy':ab,ti OR 'tumor associated fatigue':ab,ti OR 'CRF':ab,ti OR 'treatment-related fatigue':ab,ti OR 'tired*':ab,ti OR 'asthenia':ab,ti \| \| #9 \| #7 OR #8 \| \| #10 \| 'randomized controlled trial'/exp OR 'controlled clinical trial'/exp \| \| #11 \| 'randomized controlled trial':ab,ti OR 'controlled clinical trial':ab,ti OR 'randomized':ab,ti OR 'placebo':ab,ti OR 'clinical trials as topic' OR 'randomly':ab,ti OR 'trial':ab,ti \| \| #12 \| #10 OR #11 \| \| #13 \| **#3** AND **#6** AND **# 9** AND **#12** \| | 307 |
| **Cochrane Library** | \| #1 \| #1 MeSH descriptor: [Neoplasms] explode all trees \| \| --- \| --- \| \| #2 \| (Cancer or tumor or carcinoma or oncology or neoplasm or neoplasia* or malignanc* or malignant neoplasm or neoplasm*,malignant):ti,ab,kw \| \| #3 \| #1 or #2 \| \| #4 \| MeSH descriptor: [Medicine, Chinese Traditional] explode all trees \| \| #5 \| MeSH descriptor: [Acupuncture] explode all trees \| \| #6 \| MeSH descriptor: [Acupuncture, Ear] explode all trees \| \| #7 \| MeSH descriptor: [Electroacupuncture] explode all trees \| \| #8 \| MeSH descriptor: [Moxibustion] explode all trees \| \| #9 \| MeSH descriptor: [Acupressure] explode all trees \| \| #10 \| MeSH descriptor: [Cupping Therapy] explode all trees \| \| #11 \| MeSH descriptor: [Transcutaneous Electric Nerve Stimulation] explode all trees \| \| #12 \| MeSH descriptor: [Qigong] explode all trees \| \| #13 \| MeSH descriptor: [Tai Ji] explode all trees \| \| #14 \| MeSH descriptor: [Complementary Therapies] explode all trees \| \| #15 \| (Medicine, Chinese Traditional or Traditional Chinese Medicine or TCM or Chinese External Medicine or Acupuncture or electric-acupuncture or Electro-acupuncture or Electroacupuncture or needling or moxibustion or Auricular therapy or acupoint massage or acupoint injection or point application therapy or catgut embedding therapy or acupoint embedding or transcutaneous electric nerve stimulation or auricular acupuncture or ear acupoint or ear point bean-pressing or auriculotherapy or Tuina or cupping or foot bath or Fuming Therapy or fumigation or Traditional Chinese medicine emotional care or TCM emotional therapy or five elements music therapy or Qigong therapy or qigong or taijiquan or taichi or shadow boxing or baduanjin or Yijinjing or Traditional Chinese Exercises or TCM exercise therapies or complementary therapies or Traditional Chinese medicine sticking):ti,ab,kw \| \| #16 \| #4 or #5 or #6 or #7 or #8 or #9 or #10 or #11 or #12 or #13 or #14 or #15 \| \| #17 \| MeSH descriptor: [Fatigue] explode all trees \| \| #18 \| (Fatigue or lassitude or cancer fatigue or cancer related fatigue or Cancer-related fatigue or cancer-induced fatigue or Chemotherapy-related fatigue or Radiotherapy-related fatigue or fatigue after chemotherapy or tumor associated fatigue or CRF or treatment-related fatigue or tired* or asthenia):ti,ab,kw \| \| #19 \| #17 or #18 \| \| #20 \| MeSH descriptor: [Randomized Controlled Trial] explode all trees \| \| #21 \| (Randomized Controlled Trial or controlled clinical trial or randomized or placebo or clinical trials as topic or randomly or trial):ti,ab,kw \| \| #22 \| #20 or #21 \| \| #23 \| #3 or #16 or #19 or #22 \| | 633 |
| **Web of Science** | \| #1 \| TS=(Cancer OR tumor OR carcinoma OR oncology OR neoplasm OR neoplasia* OR malignanc* OR "malignant neoplasm" OR "neoplasm*,malignant") \| \| --- \| --- \| \| #2 \| TS=("Medicine, Chinese Traditional" OR "Traditional Chinese Medicine" OR TCM OR "Chinese External Medicine" OR Acupuncture OR electric-acupuncture OR Electro-acupuncture OR Electroacupuncture OR needling OR moxibustion OR "Auricular therapy" OR "acupoint massage" OR "acupoint injection" OR "point application therapy" OR "catgut embedding therapy" or "acupoint embedding" OR "transcutaneous electric nerve stimulation" OR "auricular acupuncture" OR "ear acupoint" or "ear point bean-pressing" OR auriculotherapy OR Tuina OR cupping OR "foot bath" OR "Fuming Therapy" OR fumigation OR "Traditional Chinese medicine emotional care" OR "TCM emotional therapy" OR "five elements music therapy" OR "Qigong therapy" OR qigong OR taijiquan OR taichi OR "shadow boxing" OR baduanjin OR Yijinjing OR "Traditional Chinese Exercises" OR "TCM exercise therapies" OR "complementary therapies" OR "Traditional Chinese medicine sticking") \| \| #3 \| TS=(Fatigue OR lassitude OR "cancer fatigue" OR "cancer related fatigue" OR "Cancer-related fatigue" OR "cancer-induced fatigue" OR "Chemotherapy-related fatigue" OR "Radiotherapy-related fatigue" OR "fatigue after chemotherapy" OR "tumor associated fatigue" OR CRF OR "treatment-related fatigue" OR tired* OR asthenia) \| \| #4 \| TS=("Randomized Controlled Trial" OR "controlled clinical trial" OR randomized OR randomly OR placebo OR "clinical trials as topic" OR trial ) \| \| #5 \| #1 and #2 and #3 and #4 \| | 541 |
| **Chinese Biomedical Literature Database** | \| #1 \| ( "癌症"[常用字段:智能] OR "癌"[常用字段:智能] OR "瘤"[常用字段:智能] OR "肿瘤"[常用字段:智能] OR "恶性肿瘤"[常用字段:智能] OR "白血病"[常用字段:智能] OR "恶性疾病"[常用字段:智能]) \| \| --- \| --- \| \| #2 \| ( "中医外治"[常用字段:智能] OR "针灸"[常用字段:智能] OR "针刺"[常用字段:智能] OR "灸法"[常用字段:智能] OR "灸"[常用字段:智能] OR "温针灸"[常用字段:智能] OR "艾灸"[常用字段:智能] OR "电针"[常用字段:智能] OR "揿针"[常用字段:智能] OR "穴"[常用字段:智能] OR "穴位"[常用字段:智能] OR "穴位按压"[常用字段:智能] OR "穴位按摩"[常用字段:智能] OR "穴位注射"[常用字段:智能] OR "穴位贴敷"[常用字段:智能] OR "穴位埋线"[常用字段:智能] OR "穴位热熨"[常用字段:智能] OR "经皮穴位电刺激"[常用字段:智能] OR "耳穴压贴"[常用字段:智能])OR "耳穴按摩"[常用字段:智能] OR "耳穴"[常用字段:智能] OR "耳掀针"[常用字段:智能] OR "中医传统功法"[常用字段:智能] OR "八段锦"[常用字段:智能] OR "太极拳"[常用字段:智能] OR "五禽戏"[常用字段:智能] OR "六字诀"[常用字段:智能] OR "易筋经"[常用字段:智能] OR "药枕疗法"[常用字段:智能] OR "中药足浴"[常用字段:智能] OR "中药熏蒸"[常用字段:智能] OR "中药熏洗"[常用字段:智能] OR "按摩"[常用字段:智能] OR "推拿"[常用字段:智能] OR "拔罐"[常用字段:智能] OR "五行音乐疗法"[常用字段:智能] OR "中医情志护理"[常用字段:智能]) \| \| #3 \| ( "癌因性疲乏"[常用字段:智能] OR "癌症相关性疲乏"[常用字段:智能] OR "肿瘤相关性疲乏"[常用字段:智能] OR "癌性疲乏"[常用字段:智能] OR "CRF"[常用字段:智能] OR "疲乏"[常用字段:智能] OR "乏力"[常用字段:智能] OR "疲劳"[常用字段:智能] OR "劳累"[常用字段:智能]) \| \| #4 \| #1 AND #2 AND #3 \| | 895 |
| **WANFANG Database** | 主题:(癌症 or 癌 or 瘤 or 肿瘤 or 恶性肿瘤 or 白血病 or 恶性疾病) and 主题:(中医外治 or 针灸 or 针刺 or 灸法 or 灸 or 温针灸 or 艾灸 or 电针 or 揿针 or 穴 or 穴位 or 穴位按压 or 穴位按摩 or 穴位注射 or 穴位贴敷 or 穴位埋线 or 穴位热熨 or 经皮穴位电刺激 or 耳穴压贴 or 耳穴按摩 or 耳穴 or 耳掀针 or 中医传统功法 or 八段锦 or 太极拳 or 五禽戏 or 六字诀 or 易筋经 or 药枕疗法 or 中药足浴 or 中药熏蒸 or 中药熏洗 or 按摩 or 推拿 or 拔罐 or 五行音乐疗法 or 中医情志护理) and 主题:(癌因性疲乏 or 癌症相关性疲乏 or 肿瘤相关性疲乏 or 癌性疲乏 or CRF or 疲乏 or 乏力 or 疲劳 or 劳累) | 404 |
| **China National Knowledge Internet** | SU=('癌症' + '癌 ' + '瘤' + '肿瘤' + '恶性肿瘤' + '白血病' + '恶性疾病') * ('中医外治' + '针灸' + '针刺' + '灸法' + '灸' + '温针灸' + '艾灸' + '电针' + '揿针' + '穴' + '穴位' + '穴位按压' + '穴位按摩' + '穴位注射' + '穴位贴敷' + '穴位埋线' + '穴位热熨' + '经皮穴位电刺激' + '耳穴压贴' + '耳穴按摩' + '耳穴' + '耳掀针' + '中医传统功法' + '八段锦' + '太极拳' + '五禽戏' + '六字诀' + '易筋经' + '药枕疗法' + '中药足浴' + '中药熏蒸' + '中药熏洗' + '按摩' + '推拿' + '拔罐' + '五行音乐疗法' + '中医情志护理') * ('癌因性疲乏' + ' 癌症相关性疲乏' + '肿瘤相关性疲乏' + '癌性疲乏' + 'CRF' + '疲乏' + '乏力' + '疲劳' + '劳累') | 470 |
| **VIP Database** | (M=癌症 OR M=癌 OR M=瘤 OR M=肿瘤 OR M=恶性肿瘤 OR M=白血病 OR M=恶性疾病) AND (M=中医外治 OR M=针灸 OR M=针刺 OR M=灸法 OR M=灸 OR M=温针灸 OR M=艾灸 OR M=电针 OR M=揿针 OR M=穴 OR M=穴位 OR M=穴位按压 OR M=穴位按摩 OR M=穴位注射 OR M=穴位贴敷 OR M=穴位埋线 OR M=穴位热熨 OR M=经皮穴位电刺激 OR M=耳穴压贴 OR M=耳穴按摩 OR M=耳穴 OR M=耳掀针 OR M=中医传统功法 OR M=八段锦 OR M=太极拳 OR M=五禽戏 OR M=六字诀 OR M=易筋经 OR M=药枕疗法 OR M=中药足浴 OR M=中药熏蒸 OR M=中药熏洗 OR M=按摩 OR M=推拿 OR M=拔罐 OR M=五行音乐疗法 OR M=中医情志护理 ) AND (M=癌因性疲乏 OR M=肿瘤相关性疲乏 OR M=癌性疲乏 OR M=CRF OR M=疲乏 OR M=乏力 OR M=疲劳 OR M=劳累) | 253 |
