# Supplementary material for: Efficacy of traditional Chinese medicine external therapy on cancer-related fatigue: a systematic review and network meta-analysis
Source: Front Oncol. 2026 Apr 22;16:1806355. doi: 10.3389/fonc.2026.1806355 (PMC13143725; doi:10.3389/fonc.2026.1806355)
Supplement: Supplementary file 8 [file Table2.docx]

**Supplementary table 2** Detailed explanation for each eligible intervention.

| **elgible intervention** | **detailed explanation** |
| --- | --- |
| Acupuncture | This involves the insertion of thin needles into specific points on the body to balance the flow of Qi (energy) and promote healing. Acupuncture is believed to stimulate the nervous system, releasing endorphins and other neurochemicals that can help reduce pain and fatigue. |
| Acupressure | A non-invasive technique that applies pressure to specific acupoints on the body using fingers, palms, or elbows. This method aims to relieve tension and improve energy flow, potentially alleviating symptoms of CRF. |
| Traditional Chinese Exercises | This category includes practices such as Tai Chi, Baduanjin, and Qi Gong, which combine gentle physical movements, breathing techniques, and meditation. These exercises promote physical fitness, mental well-being, and relaxation, all of which can contribute to reduced fatigue. |
| Transcutaneous Acupoint Electrical Stimulation (TAES) | This technique involves using electrical impulses to stimulate acupoints without needles. It is thought to activate similar pathways as acupuncture, potentially providing relief from CRF. |
| Moxibustion | A traditional treatment that involves burning moxa (a dried mugwort herb) near specific acupuncture points or on the skin. Moxibustion is believed to warm the meridians and enhance the flow of Qi, which may help alleviate fatigue and improve energy levels. |
| Auricular Acupressure | This technique applies pressure to specific points on the ear, which correspond to various body parts and functions. It aims to regulate body systems and relieve symptoms associated with CRF. |
| Chinese Medicine Foot Bath | This involves soaking the feet in herbal-infused water, which is thought to promote relaxation and improve circulation. It may also help detoxify the body and enhance overall well-being. |
| Chinese Medicine Emotional Care | A holistic approach that integrates emotional support and traditional practices to address psychological aspects of fatigue. First, assess the patient's emotional state through observation and conversation, identifying any signs of stress, anxiety, or depression. Create a calming environment by minimizing distractions and using soothing elements like soft lighting and gentle music. Engage the patient in relaxation techniques such as guided meditation or deep breathing exercises to promote emotional well-being. Incorporate TCM principles by discussing dietary recommendations, herbal remedies, and lifestyle changes that can support emotional health. Additionally, encourage the practice of Tai Chi or Qi Gong to enhance emotional balance and physical well-being. Finally, maintain regular follow-ups to monitor the patient's progress and adjust care strategies as needed, fostering a supportive and empathetic therapeutic relationship. |
| Acupoint Injection | This method involves injecting therapeutic substances (e.g., traditional Chinese herbal extracts) into specific acupoints. It aims to enhance the effectiveness of treatment by directly delivering medicinal properties to the targeted areas. |
| Acupoint Application | This involves applying herbal pastes or plasters to specific acupoints on the body, promoting localized healing effects that may alleviate CRF symptoms. |
| Auricular Press Needle | A technique that uses small needles placed on specific ear acupoints to provide continuous stimulation. This method is often used for self-care and can help manage fatigue. |
| warming needle | A combined approach that uses both acupuncture and moxibustion, leveraging the benefits of both modalities to enhance treatment outcomes for CRF.first, prepare the treatment area and ensure the patient is comfortable. Select appropriate acupoints based on the patient's condition, then insert sterile acupuncture needles into these points. Light a moxa stick and hold its burning end approximately 1-2 inches above the needles, allowing the heat to penetrate the skin while monitoring the patient's comfort. Maintain this for about 10-20 minutes, ensuring the heat is soothing. After the session, extinguish the moxa stick, remove the needles, and provide post-treatment care instructions to the patient. |
| Acupoint Hot Ironing | This involves the application of heated tools on acupoints, providing warmth and stimulation. It is believed to enhance blood circulation and relieve muscle tension, potentially alleviating fatigue. |
