# Supplementary material for: Efficacy of traditional Chinese medicine external therapy on cancer-related fatigue: a systematic review and network meta-analysis
Source: Front Oncol. 2026 Apr 22;16:1806355. doi: 10.3389/fonc.2026.1806355 (PMC13143725; doi:10.3389/fonc.2026.1806355)
Supplement: Supplementary file 9 [file Table3.docx]

**Supplementary table 3** Literature excluded from full-text review.

| **Title of Literature** | **The first author** | **Journal Title** | **Reasons for exclusion** |
| --- | --- | --- | --- |
| Acupuncture Care for Breast Cancer Patients During Chemotherapy: A Feasibility Study | Sarah Price | Integrative cancer therapies | Not RCT |
| Clinical Observation of Wen Moxibustion Therapy Cancer - related Fatigue of Advanced Cancer (in Chinese) | Xiaoyan Tan | China Journal of Chinese Medicine | Outcome not match |
| Acupuncture for nonpalliative radiation therapy-related fatigue: feasibility study | [Jun James Mao](https://pubmed.99885.net/?term=Mao+JJ&cauthor_id=19476739) | Journal of the Society for Integrative Oncology | Not RCT |
| The therapeutic effect of moxibustion on cancer-related fatigue in elderly patients with advanced tumors (in Chinese) | Jili Yang | Chinese Journal of Gerontology | Outcome not match |
| Clinical characteristics analysis of cancer-related fatigue and observation of the effect of moxibustion treatment | Lei Yu | Guide of China Medicine | Outcome not match |
| Study on auricular acupressure to relieve cancer- related fatigue in breast cancer patients accepting chemotherapy after surgury (in Chinese) | Lanfang Yu | Chinese General Practice Nursing | Outcome not match |
| Acupuncture for cancer-related fatigue in patients with breast cancer: a pragmatic randomized controlled trial | [Alexander Molassiotis](https://pubmed.99885.net/?term=Molassiotis+A&cauthor_id=23109700) | Journal of clinical oncology | No available data for analysis |
| A randomized, controlled trial of acupuncture self-needling as maintenance therapy for cancer-related fatigue after therapist-delivered acupuncture | [A Molassiotis](https://pubmed.99885.net/?term=Molassiotis+A&cauthor_id=23436910) | Annals of oncology | No available data for analysis |
| Evaluation of an acupuncture service in oncology | J Salmon | Journal of Radiotherapy in Practice | Not RCT |
| The effect of moxibustion on cancer-related fatigue in lung cancer chemotherapy patients | Chaoyang Liu | Traditional Chinese Medicine Rehabilitation | Outcome not match |
| Clinical Observation on 28 Cases of Cancer related Fatigue Caused by Chemotherapy for breast cancer Treated with Foot Bath of Yiqi Chinese Medicine (in Chinese) | Yongping Xu | Guiding Journal of Traditional Chinese Medicine and Pharmacy | Outcome not match |
| Study on influence of Baduanj in exercise on cancer chemotherapy patients with cancer related fatigue (in Chinese) | Minning Xiu | Chinese General Practice Nursing | Outcome not match |
| Pilot Randomized Controlled Trial of Auricular Point Acupressure to Manage Symptom Clusters of Pain, Fatigue, and Disturbed Sleep in Breast Cancer Patients | [Chao Hsing Yeh](https://pubmed.99885.net/?term=Yeh+CH&cauthor_id=26390073) | Cancer nursing | No available data for analysis |
| Clinical study on acupuncture treatment of cancer-related fatigue in 30 patients with breast cancer (in Chinese) | Jun chen | Jiangsu Journal of Traditional Chinese Medicine | Outcome not match |
| The effect of traditional Chinese medicine foot bath on cancer-related fatigue in patients with gastrointestinal tumors undergoing chemotherapy (in Chinese) | Guiping Zhu | Chinese Journal of Modern Drug Application | Outcome not match |
| The effect of ear acupressure on cancer-related fatigue in cancer chemotherapy patients (in Chinese) | Juxiang Ding | Modern Nurse | Outcome not match |
| Prophylactic acupuncture treatment during chemotherapy with breast cancer: a randomized pragmatic trial with a retrospective nested qualitative study | [Benno Brinkhaus](https://pubmed.99885.net/?term=Brinkhaus+B&cauthor_id=31520284) | Breast cancer research and treatment | No available data for analysis |
| Moxibustion for treating cancer- related fatigue: A multicenter, assessor- blinded, randomized controlled clinical trial | Kyungsun Han | Cancer medicine | No available data for analysis |
| Acupuncture for postchemotherapy fatigue: a phase II study | [Andrew J Vickers](https://pubmed.99885.net/?term=Vickers+AJ&cauthor_id=15117996) | Journal of clinical oncology | Not RCT |
| Efficacy of saam acupuncture treatment on improvement of immune cell numbers in cancer patients: a pilot study | [Dae-Joon Kim](https://pubmed.99885.net/?term=Kim+DJ&cauthor_id=25417404) | Journal of traditional Chinese medicine | Not RCT |
| Feasibility Analysis on Space-time Acupuncture Program of Eight Methods of Sacred Tortoise in the Intervention of Postoperative Chemotherapy Fatigue in Breast Cancer | Zheng Zuo | World Chinese Medicine | Not RCT |
| Cancer-related fatigue | Patrick C.Stone | [European Journal of Cancer](https://www.sciencedirect.com/journal/european-journal-of-cancer) | Not RCT |
| Auricular Acupuncture During Chemotherapy Infusion in Breast Cancer Patients: A Feasibility Study | [EunMee Yang](https://pubmed.99885.net/?term=Yang+E&cauthor_id=35238615) | Journal of integrative and complementary medicine | Not RCT |
| The therapeutic effect of moxibustion on cancer-related fatigue in elderly patients with advanced tumors (in Chinese) | Wei Qi | Medical Aesthetics and Cosmetology | Lack details of the intervention |
| Randomized controlled study on acupuncture treatment of cancer-related fatigue in convalescent patients with breast cancer (in Chinese) | Hong Pan | Yiyao Qianyan | Lack details of the intervention |
| The impact of cancer-related fatigue on patients with malignant tumors during radiotherapy (in Chinese) | Yingzi Gw | Oriental Tonic Diet | Lack details of the intervention |
| The effect of ear acupressure on cancer-related fatigue in cancer chemotherapy patients (in Chinese) | Long Hao | Friends of Health | Lack details of the intervention |
| Clinical observation on acupuncture assisted treatment of cancer-related fatigue (in Chinese) | Juan Jiang | Journal of Practical Traditional Chinese Medicine | Lack details of the intervention |
| Effect of moxibustion on relieving cancer-related fatigue in patients with breast cancer undergoing chemotherapy (in Chinese) | Yunyue Lin | Yiyao Qianyan | Lack details of the intervention |
| Application effect of moxibustion therapy nursing intervention in postoperative gastric cancer patients (in Chinese) | Jing Xiao | Henan Medical Research | Lack details of the intervention |
| The impact of moxibustion on the quality of life of patients with advanced liver cancer (in Chinese) | Kun Zhang | Health Must Read | Lack details of the intervention |
| The effect of inserting needles into the bilateral Zusanli area on cancer-related fatigue in patients with malignant tumors during treatment (in Chinese) | Shufen Huang | Diet Health | Lack details of the intervention |
| Study on the effect of Ba Duan Jin on cancer-related fatigue in patients with lung cancer undergoing chemotherapy (in Chinese) | Qin Yue | self health care | Lack details of the intervention |
| Analysis of the therapeutic effect of moxibustion on cancer-related fatigue in elderly patients with advanced tumors (in Chinese) | Yi Zhang | Medical Dietary Therapy and Health | Lack details of the intervention |
