# Supplementary material for: Efficacy of traditional Chinese medicine external therapy on cancer-related fatigue: a systematic review and network meta-analysis
Source: Front Oncol. 2026 Apr 22;16:1806355. doi: 10.3389/fonc.2026.1806355 (PMC13143725; doi:10.3389/fonc.2026.1806355)
Supplement: Supplementary file 10 [file Table4.docx]

**Supplementary table 4** Basic characteristics of the eighty-seven studies.

|  | **Author, year** | **Country** | **Study design** | **Sample size (N);**  **population** | **Mean age (year)** | **Intervention, N** | **Control, N** | **Outcome**  **measure**  **tools** | **funding** |
| --- | --- | --- | --- | --- | --- | --- | --- | --- | --- |
| 1 | Molassiotis et al,^32^2007 | UK | Parallel RCT;  three-arm | 47,mixed cancer | Total :53.4 | Acupuncture, 15  Intensity: 2 weeks, 3 times per week, 20 min per time;  Acupoints:LI4, SP6 , ST36  Acupressure,16  Intensity: 2 weeks, once per day,  stimulate each point for 3 min;  Acupoints:LI4, SP6 , ST36 | Sham acupressure,16  Using 3 sham acupoints. | MFI | Ministry of Science and Technology, Taiwan (MOST 110-2628-B-038-017) |
| 2 | Deng  et al,^31^2013 | China | Parallel RCT | 97,mixed cancer | Intervention:  54.0  Control: 53.0 | Acupuncture, 47  Intensity: 6 weeks, once per week, 20 min per time;  Acupoints:CV6,CV4 ,KI3,ST36,SP6,LI11,HT6 | Sham acupuncture, 50  Sham needles (nonpenetrating retractable needles) were placed in sham acupoints a few millimeters from the real acupuncture points. | BFI | United States Department of Health & Human Services National Institutes of Health (NIH) - USA NIH National Cancer Institute (NCI)  NCI NIH HHS |
| 3 | Balk  et al,^32^2009 | USA | Parallel pilot  RCT | 27,NR | Intervention:  54.0  Control: 53.7 | Acupuncture, 16  Intensity: 4-6 weeks, 1-2 per week, 30 min per time;  Acupoints:Ki3, Sp6, LI4, St36,Ren6/CV6 | Sham acupuncture, 11  Sham needles (nonpenetrating retractable needles) were placed in sham acupoints . | FACIT-F | National Cancer Institute (R21 CA098659-01A2). |
| 4 | Oh  et al,^33^2010 | Australia | Parallel RCT | 162,mixed cancer | Intervention:  60.1  Control: 59.9 | Traditional Chinese exercises(Qi Gong),79  Intensity: 10 weeks, 2 per week, 90 min per time;  Details: 15-min discussion of health issues, 30-min gentle stretching and body movement in standing postures , 15-min movement in seated posture,30-min meditation including breathing exercises | Usual care, 83 | FACT-F | University of Sydney Cancer Research Fund |
| 5 | Sun  et al,^34^ 2011 | China | Parallel RCT | 60,NR | Intervention:  39.2  Control: 37.6 | Transcutaneous acupoint electrical stimulation,30  Intensity: 1 weeks, once per day, 20 min per time;2/100Hz,9-12mA  Acupoints:SP10,ST36,SP6 | Usual care, 30 | PRFS | 2010 Hospital level Project of Longhua Hospital Affiliated to Shanghai University of Traditional Chinese Medicine (2010YZ30) |
| 6 | Lu  et al,^35^ 2012 | China | Parallel pilot  RCT | 21,Ovarian cancer | Intervention:  50.8  Control: 50.0 | Acupuncture, 11  Intensity: 4 weeks, 2-3 per week, 30 min per time;  Acupoints:SP10,ST36,SP6,K3,LR3,PC6,LI11,LI4 | Sham acupuncture, 10  The sham acupuncture protocol used 5 nonacupuncture  points. | EORTC QLQ-C30 | National Center for Complementary and Alternative Medicine (NCCAM).  (1U19AT002022-01) |
| 7 | Chen  et al, ^36^2013 | China | Parallel RCT | 96,Breast cancer | Intervention:  45.3  Control: 44.7 | Traditional Chinese exercises(Qi Gong),49  Intensity: 4-5 weeks, 5 per week, 40 min per time;  Details: 13-min preparation exercise consisted; 18-min main exercise;9-min ending exercise | Wait list, 47 | BFI |  |
| 8 | Jiang  et al,^37^ 2013 | China | Parallel RCT | 60,Lung cancer | Intervention:  64.4  Control: 65.6 | Traditional Chinese exercises(Tai Chi),30  Intensity: 30 days, 2 per day, 30 min per time; | Usual care, 30 | BFI | Research Project of Longhua Hospital Affiliated to Shanghai University of Traditional Chinese Medicine  (2011YZ24) |
| 9 | Loh  et al,^38^ 2014 | Malaysia | Parallel RCT;  three-arm | 64,Breast cancer | NR | Traditional Chinese exercises(Qi Gong),49  Intensity: 8 weeks, 2 per week, 90 min per time; | Usual care, 32 | FACIT-F | NA |
| 10 | Mao  et al, ^39^2014 | USA | Parallel RCT;  three-arm | 67,Breast cancer | Intervention:  57.5  Control1:60.9  Control2: 60.6 | Acupuncture, 22  Intensity: 2 weeks, 2 per week; | Sham acupuncture, 22  Sham needles (nonpenetrating retractable needles) were placed in sham acupoints .  Wait list, 23 | BFI | NIH/NCCAM R21  NCCAM K23 AT004112 award |
| 11 | Tang  et al, ^40^2014 | China | Parallel pilot  RCT | 40,Lung cancer | Intervention:  54.8  Control: 66.1 | Acupressure,24  Intensity: 20 weeks, once per day,  stimulate each point for 1 min;  Acupoints:LI4, SP6 , ST36 | Sham acupressure, 16 | TFRS | National Science Council in  Taiwan ( NMRPD180821) |
| 12 | Guo  et al, ^41^2014 | China | Parallel RCT | 80,Gynecological cancer | Intervention:  51  Control: 53 | Acupuncture+Moxibustion,40  Intensity: 3 weeks, 3 per week,  20 min per time;  Acupuncture Acupoints:RN12,PC6,ST36,SP6,CV6,CV4,BL23,BL18  Moxibustion Acupoints:ST36,CV6,CV4 | Usual care, 40 | BFI | China Postdoctoral Science Foundation Project  (20110491102)  Harbin Science and Technology Innovation Talent Research Special Fund Project  ( 2-11RFQYS087) |
| 13 | Larkey  et al,^42^ 2015 | USA | Parallel RCT | 86,Breast cancer | Intervention:  57.7  Control: 60.9 | Traditional Chinese exercises(Qi Gong/Tai Chi),44  Intensity: 12 weeks, 5 per week, 30 min per time; | Sham (Tai Chi/Qi Gong),42  perception that the intervention was “like Tai Chi or Qi gong” , while significantly different for perception of meditative focus (as intended). | FSI | National Center for Complementary and Alternative Medicine  and Office of Women’s Health (5 U01 AT002706-03)  Arizona Cancer Center Support Grant(P50 CA023074) |
| 14 | Zhang  et al,^43^ 2015 | China | Parallel RCT | 152,Rectal cancer | NA | Chinese medicine foot bath,76  Intensity: 1 weeks, once a day,  30 min per time;  Herbal foot bath:1000ML,40℃-50℃. | Usual care, 76 | PFS | Project of Zhejiang Provincial Administration of Traditional Chinese Medicine  (2012zB050)  Zhejiang Province Traditional Chinese Medicine Administration Science and Technology Bureau Project (2011zA033) |
| 15 | Mao  et al,^44^ 2016 | China | Parallel RCT | 78,Lung cancer | Intervention:  59.1  Control: 59.7 | Moxibustion,39  Intensity: 4 weeks, 3 per day,  20 min per time;  Acupoints:ST36,CV6,CV4 | Sham moxibustion, 39  the laser source was cutting off when the in strument was turned on. | BFI | National Natural Science Foundation of China  (81320108028)  National Basic Research Program of China (2015CB554505)  the Key Program of the  State Administration of Traditional Chinese Medicine of The People’s Republic of China (ZYSNXD-CC-ZDXK-07). |
| 16 | Molassiotis  et al,^45^ 2021 | Vietnam | Parallel RCT | 156,mixed cancer | Intervention:  57.6  Control: 56.0 | Traditional Chinese exercises(Qi Gong),78  Intensity: 6 weeks, 4 per week, 30 min per time;  The training involved a series of simple, repeated practices including body posture/movement, breathing practice, and meditation performed in synchrony. | Usual care, 78 | FACIT | NA |
| 17 | Zick  et al,^46^ 2016 | USA | Parallel RCT | 190,Breast cancer | Intervention:  60.8  Control: 61.0 | Acupressure,94  Intensity: 6 weeks, once per day,  stimulate each point for 3 min;  Acupoints:GV20,CV6,LI4,ST36,SP6,KD3. | Usual care, 96 | BFI | National Institutes of Health.  (R01 CA151445)  (2UL1 TR000433-06) |
| 18 | Yeh  et al, ^47^2016 | China | Parallel RCT | 108,lymphoma | Total :59.7 | Traditional Chinese exercises(Qi Gong),54  Intensity: 3 weeks, 2-3 per week, 15-60 min per time; | Usual care, 54 | VAS | NA |
| 19 | Ding  et al,^48^ 2016 | China | Parallel RCT | 86,leukaemia | Intervention:  46  Control: 46 | TCM emotional care,43  Intensity: NR  Details:  Relaxation through music; complying with reasonable patient needs; alleviating negative patient emotions through communication and venting; reducing patient anxiety by diverting attention; providing a good nurturing environment for patients; establishing patient awareness of the disease; meditation, QiGong; acupoint massage. | Usual care, 43 | FQ | Nursing project of Wuxi Hospital Management Center  (YGZXHl313) |
| 20 | He  et al,^49^ 2016 | China | Parallel RCT | 64,Breast cancer | Total :48.6 | Traditional Chinese exercises(Baduanjin),31  Intensity: 4 weeks, 2 per day, 15-20 min per time; | Usual care, 33 | BFI | Zhejiang Province Traditional Chinese Medicine Science and Technology Plan Project (2013ZA062) |
| 21 | Li  et al, ^50^2016 | China | Parallel RCT | 80,mixed cancer | Intervention:  51  Control: 53 | Acupuncture,40  Intensity: 2 weeks, once a day,  30 min per time;  Acupoints:DU2,RN12,CV6,SP10,ST36,SJ5 | Usual care, 40 | MFI | NA |
| 22 | Ma  et al,^51^ 2016 | China | Parallel RCT | 32,mixed cancer | NR | Acupoint injection,13  Intensity: 3 weeks, 1 per week;  Acupoints:ST36  Details:  Injecting Astragalus injection into bilateral Zusanli acupoints. | Usual care, 19 | EORTC QLQ-C30 | Ministry of Science and Technology's Science and Technology Assistance Project for Developing Countries  ( KY201302010) |
| 23 | Su  et al, ^52^2016 | China | Parallel RCT | 64,mixed cancer | Intervention:  60  Control: 62 | Acupuncture,40  Intensity: 2 weeks, once a day,  30 min per time;  Acupoints:KI3,ST36,SP10,CV6,CV4,Xuanzhong. | Usual care, 40 | PRFS | NA |
| 24 | Wu  et al, ^53^2016 | China | Parallel RCT | 120,lung cancer | Intervention:  52.6  Control: 53.3 | Moxibustion,60  Intensity: 3 weeks, once a day,  30 min per time;  Acupoints:CV8,KI1,CV6,BL13 | Usual care, 60 | PRFS | NA |
| 25 | Zhang  et al, ^54^2016 | China | Parallel RCT | 44,mixed cancer | Intervention:  55  Control: 62 | Moxibustion,22  Intensity: 10 days, once a day;  Acupoints:ST36 | Usual care, 22 | EORTC QLQ-C30 | Research Project of Nanjing Health Bureau, Jiangsu Province  (YKKO12059) |
| 26 | Cheng  et al, ^55^2017 | China | Parallel RCT | 28,lung cancer | Intervention:  58  Control: 62 | Acupuncture,14  Intensity: 4 weeks, 2 per week ,  45 min per time;  Acupoints:LI4, Ren6, St36, KI3, Sp6 | Sham acupuncture, 14 | BFI | Comprehensive and Integrative Medicine Institute (CIMI) National Natural Science Foundation of China  (No. 81403248) |
| 27 | Chuang  et al, ^56^2017 | China | Parallel RCT | 96,lymphoma | Intervention:  55.8  Control: 64.5 | Traditional Chinese exercises(Qi Gong),48  Intensity: 3 weeks, 2-3 per day, 25 min per time; | Usual care, 48 | EORTC QLQ-C30 | NA |
| 28 | Hou  et al, ^57^2017 | China | Parallel RCT | 113,lung cancer | Intervention:  58.0  Control: 58.2 | Transcutaneous acupoint electrical stimulation,57  Intensity: 4 weeks,30 min per time;30/100 Hz ,6–15 V;  Acupoints:UB17,ST36,CV6 | Usual care, 56 | RPFS | National Nature and Science Foundation of China  ( Nur81202750) |
| 29 | McQuade  et al,^58^ 2016 | USA | Parallel RCT | 45,Prostate cancer | Intervention:  62.2  Control: 65 | Traditional Chinese exercises(Qi Gong/Tai Chi),21  Intensity: 12 weeks, 3 per week, 40 min per time;  Details: preparation exercises (6 minutes);main exercises (20 minutes);ending exercises (9 minutes) | Usual care, 24 | BFI | NCI CA129201 and  CA016672  Center for Energy Balance in Cancer Prevention |
| 30 | Wu  et al,^59^ 2017 | China | Parallel RCT  Three arm | 162,lung  cancer | Intervention:  57  Control1: 49  Control2: 56 | Transcutaneous acupoint electrical stimulation,57  Intensity: 4 weeks,2 per day;30 min per time;30/100 Hz ;  Acupoints:CV6，BL17，ST36 | Usual care, 56  Sham transcutaneous electric nerve stimulation,49  Use of false acupoints | RPFS | Shanghai Talent Development Fund Support  (201449) |
| 31 | Xu  et al, ^60^2017 | China | Parallel RCT | 80,mixed cancer | Intervention:  58.1  Control: 60.0 | Moxibustion,40  Intensity: 4 weeks, once a day,  10-20 min each point ;  Acupoints:ST36，SP10，KI3，CV6，CV4，Xuanzhong | Usual care, 40 | PFS | NA |
| 32 | Yu  et al,^61^ 2017 | China | Parallel RCT | 72,Breast cancer | Intervention:  50.2  Control: 51.4 | Acupuncture,36  Intensity: 4 weeks, 2 per week ;  Acupoints:DU20,PC6,CV6,ST36,SP6 | Sham acupuncture,36  Use of false acupoints  Superficial needling technique | PFS | Traditional Chinese Medicine Science and Technology Fund Project of Beijing Administration of Traditional Chinese Medicine  (JJ201 1-04)  Beijing Hospital Management Bureau Youth Talent Training "Young Seedlings" Program Project  (QML20150903). |
| 33 | Yuan  et al, ^62^2017 | China | Parallel RCT | 64,Colorectal cancer | Intervention:  61.2  Control: 60.8 | Moxibustion,32  Intensity: 30 days, once a day ;  Acupoints:GV3,GV15,GV9,GV16,GV4. | Usual care, 32 | RPFS | Traditional Chinese Medicine Technology Project of Chongqing Health and Family Planning Commission (zy201602028) |
| 34 | Chen  et al,^63^ 2018 | China | Parallel RCT | 64,mixed cancer | Intervention:  60.3  Control: 59.8 | TCM emotional care,32  Intensity: NR  Details:  Relaxation through music; complying with reasonable patient needs; alleviating negative patient emotions through communication and venting; reducing patient anxiety by diverting attention; acupoint massage. | Usual care, 32 | CFS | NA |
| 35 | Deng  et al, ^64^2018 | China | Parallel RCT | 60,mixed cancer | Intervention:  48.9  Control: 50.1 | Acupuncture,30  Intensity: 4 weeks, 2 per week,30 min per time ;  Acupoints:LI4,PC6,LR3,PC9,GB34,ST40,SP6,GV20 | Usual care, 30 | EORTC QLQ-C30 | Research Project of Nanjing Municipal Health Bureau (LH174-3) |
| 36 | Han  et al, ^65^2018 | China | Parallel RCT | 120,mixed cancer | Intervention:  48.9  Control: 60.3 | Acupoint application,60  Intensity: 3 weeks,once a day;  Astragalus-Atractylodes Acupoint Plaster：30g Astragalus, 15gWhite Atractylodes,15Atractylodes, 10g borneol.  Acupoints:CV8,BL13,ST36,SP6 | Usual care, 60 | BFI | Zhejiang Province Traditional Chinese Medicine Science and Technology Plan Project (2017ZA019) |
| 37 | Jiang  et al,^66^ 2018 | China | Parallel RCT | 70,Breast cancer | Intervention:  51.3  Control: 52.1 | Moxibustion,35  Intensity: 3 weeks ;  Acupoints:BL17,BL1 | Usual care, 35 | RPFS | Research Project of Guangdong Provincial Administration of Traditional Chinese Medicine (20161131) |
| 38 | Liang  et al,^67^ 2018 | China | Parallel RCT | 53,lung cancer | Intervention:  65.7  Control: 65.4 | Moxibustion,34  Intensity: 15 days, once a day;  Acupoints:BL13,BL14,BL15,BL16,BL17,BL18. | Usual care, 29 | PFS | National Natural Science Foundation of China Youth Fund  (81704185) |
| 39 | Shao  et al, ^68^2018 | China | Parallel RCT | 80,lung cancer | Intervention:  65.7  Control: 65.4 | Auricular press needle,40  Intensity: 3 weeks, 3 per day,2 min each point;  ear Acupoints:gan,pi,shen,shenmen,jiaogan. | Usual care, 40 | RPFS | NA |
| 40 | Xu  et al, ^69^2018 | China | Parallel RCT | 40,esophagus cancer | Intervention:  62.4  Control: 64.4 | Moxibustion,20  Intensity: 24 weeks, 5 per week;  Acupoints:ST36,gaohuang | Usual care, 20 | BFI | Key Science and Technology Project of Jiangsu Provincial Administration of Traditional Chinese Medicine  (ZD201505) |
| 41 | Zhang  et al,^70^ 2019 | China | Parallel RCT | 68,Lung cancer | Intervention:  59.9  Control: 62.7 | Auricular acupressure,34  Intensity: Three cycles of chemotherapy, Change the ear patch once every 3 days,6 times for 1 course of treatment; | Usual care, 34 | CFS | Suzhou Science and Technology Bureau Project  (SYS201526) |
| 42 | Khanghah  et al, ^71^2019 | Iran | Parallel RCT  Three arm | 90,mixed  cancer | Intervention:  50  Control1: 51  Control2: 51 | Acupressure,30  Intensity: NR;  Acupoints:LI4,ST36,SP6 | Usual care, 30  Sham acupressure,30 | VAS | Guilan University of Medical Sciences  (293033037) |
| 43 | Lu  et al, ^72^2019 | China | Parallel RCT | 87,Colon cancer | Intervention:  55.6  Control: 54.6 | Traditional Chinese exercises(Qi Gong),43  Intensity: 24 weeks, 5 per week, 40 min per time; | Usual care, 43 | BFI | NA |
| 44 | Chen  et al, ^73^2019 | China | Parallel RCT | 60,mixed  cancer | Intervention:  52.6  Control: 52.7 | Moxibustion,30  Intensity: 6 weeks, Every other day,30min per time;  Acupoints:CV4,CV6 | Usual care, 30 | PFS | Science and Technology Plan Project of Taizhou City, Zhejiang Province  (1401ky30) |
| 45 | Chen  et al, ^74^2019 | China | Parallel RCT | 60,mixed  cancer | Intervention:  62.2  Control: 61.4 | Moxibustion,30  Intensity: 4 weeks, once a day,40min per time;  Acupoints:CV8 | Usual care, 30 | PFS | NA |
| 46 | Ge  et al,^75^ 2019 | China | Parallel RCT | 78,mixed  cancer | Intervention:  60.9  Control: 61.0 | Traditional Chinese exercises(Tai Chi),39  Intensity: 6 weeks, 2 per day, 30 min per time; | Usual care, 39 | PFS | NA |
| 47 | Han  et al, ^76^2019 | China | Parallel RCT | 44,breast  cancer | Intervention:  46.3  Control: 45.5 | Traditional Chinese exercises(Tai Chi),23  Intensity: 12 weeks, 2 per day, 5 per week,40 min per day; | Usual care, 21 | RPFS | Natural Science Foundation of Fujian Province Science and Technology Project ( 2016J01773)  Research Project of Chinese Medical Qigong Society  (YXQG2015019) |
| 48 | He  et al, ^77^2019 | China | Parallel RCT | 51,breast  cancer | Intervention:  50.7  Control: 51.4 | Acupoint application,26  Intensity: 1 week,once a day,3h per time;  Acupoint plaster application: 10g Prepared Aconite, 15g Dried Ginger, 15g Astragalus, 10g Cloves.  Acupoints:ST36,CV4,Shanzhong | Usual care, 25 | PFS | Beijing Traditional Chinese Medicine Technology Development Fund Project (QN2014-06) |
| 49 | Wang  et al, ^78^2019 | China | Parallel RCT | 72,Gastrointestinalt  cancer | Intervention:  52.1  Control: 52.0 | Chinese medicine foot bath,36  Intensity: , once a day,  20-30 min per time;  Herbal foot bath:1000ML,30g Achyranthes bidentata, 30g Cinnamomum cassia, 30g Angelica pubescens, 30g Lycopodium clavatum, 30g Spatholobus suberectus, 15g Artemisia argyi leaves, 15g Amomum fruit | Usual care, 36 | BFI | NA |
| 50 | Wang  et al, ^79^2019 | China | Parallel RCT | 64,Gastrointestinalt  cancer | Intervention:  52.5  Control: 52.2 | Chinese medicine foot bath,34  Intensity: 2 weeks, once a day,  30 min per time;  Herbal foot bath:400ML,12g Bupleurum, 10g Cyperus, 15g Paeonia lactiflora, 15g Atractylodes macrocephala, 6g Amomum (processed), 10g Aurantium, 20g Astragalus, 15g Codonopsis, 10g Poria, 10g Sour jujube seed, 10g Citrus peel, 12g Albizia bark, 6g Licorice. | Usual care, 30 | VAS | NA |
| 51 | Wang  et al, ^80^2019 | China | Parallel RCT | 60,lung  cancer | Intervention:  62.0  Control: 62.0 | Acupoint hot ironing,30  Intensity: 24 days,once a day,50-60℃;  Acupoints:ST36 | Usual care, 30 | BFI | Science and Technology Plan Project of Jiangxi Provincial Health Commission in 2019 (20191096) |
| 52 | Xia  et al, ^81^2019 | China | Parallel RCT | 74,Thyroid cancer | Intervention:  47.6  Control: 46.2 | Moxibustion,37  Intensity: 1 weeks, once a day,20min per time;  Acupoints:GV20 | Usual care, 37 | EORTC QLQ-C30 | Traditional Chinese Medicine Research Project of Jiangxi Provincial Health and Family Planning Commission  (20178005) |
| 53 | Xu  et al, ^82^2019 | China | Parallel RCT | 82,Cervical cancer | Intervention:  49.0  Control: 50.1 | Transcutaneous acupoint electrical stimulation,41  Intensity: 6 weeks,2 per week;30 min per time;2-100 Hz ;  Acupoints:NR | Usual care, 41 | MFI | Research Project of Shandong Provincial Health and Family Planning Commission (No. 2016HWl02) |
| 54 | Zhang  et al, ^83^  2019 | China | Parallel RCT | 73,Gastric cancer | Intervention: 54.7  Control: 45.9 | Moxibustion,38  Intensity: 6 weeks, once a day;  Acupoints:RN12,CV6,ST36,BL20,BL21. | Usual care, 35 | RPFS | NA |
| 55 | Nakano  et al, ^84^2020 | Japan | Parallel RCT | 48,mixed cancer | Total:70.0 | Transcutaneous acupoint electrical stimulation,24  Intensity: once a day;30 min per time;100 Hz ;  Acupoints:NR | Usual care, 24 | EORTC QLQ-C30 | Japan Society for the Promotion of Science KAKENHI  ( 18K10711) |
| 56 | Tao  et al,^85^ 2020 | China | Parallel RCT | 64,mixed cancer | Intervention:  61.3  Control: 63.9 | Acupuncture,32  Intensity: 1 weeks, once a day,30 min per time ;  Acupoints:CV4,ST36,SP6,KI3,PC6,Zhongfeng,shenmen,Wangu | Usual care, 32 | BFI | NA |
| 57 | Li  et al,^86^ 2020 | China | Parallel RCT | 108,mixed cancer | Intervention:  59.8  Control: 56.8 | Transcutaneous acupoint electrical stimulation,55  Intensity: 5 days,once a day;20 min per time;1-100 Hz ,6-12 mA;  Acupoints:RN12,CV6,CV4,PC6,ST36,SP6 | Usual care, 53 | RPFS | Guangdong Science and Technology Plan Project (2017ZC0201) |
| 58 | Lu  et al, ^87^2020 | China | Parallel RCT | 73,Colorectal cancer | NR | Moxibustion,31  Intensity: 2 weeks, once a day,30 min per day;  Acupoints:RN12,CV6,ST36,Shanzhong. | Usual care, 32 | PFS | Tianjin Municipal Science and Technology Commission Major Special Project for Chronic Disease Prevention and Control (17ZXMF-SY00190) |
| 59 | Ni  et al,^88^ 2020 | China | Parallel RCT | 74,Gynecological cancer | Intervention:  53  Control: 53 | Auricular acupressure,37  Intensity: 8 weeks, 2-3 per day,3-5min per time;  Ear Acupoints:gan,pi,wei,shenmen,jiaogan. | Usual care, 37 | BFI |  |
| 60 | Qin  et al, ^89^2020 | China | Parallel RCT | 67,mixed cancer | Intervention:  60.0  Control: 59.0 | Acupuncture,36  Intensity: 4 weeks, once a day,5 per week,15 min per time ;  Acupoints:GV20,CV4,CV6,GB20,ST36,SP6. | Usual care, 31 | FACT-F | Guangdong Provincial Bureau of Traditional Chinese Medicine Construction of Traditional Chinese Medicine Strong Province Research Project  (20152110) |
| 61 | Su  et al,^90^ 2020 | China | Parallel RCT | 111,Lung cancer | Intervention:  52.0  Control: 54.0 | Moxibustion,56  Intensity: 2 weeks, once a day,30 min per day;  Acupoints:UB20,UB21 | Usual care, 55 | BFI | NA |
| 62 | Xu  et al, ^91^2020 | China | Parallel RCT | 92,Gastrointestinal  cancer | Intervention:  61.5  Control: 61.5 | Traditional Chinese exercises(Baduanjin),46  Intensity: 24 weeks,5 per week,25-30 min per time; | Usual care, 46 | RPFS | Research Project of Wuxi Health and Family Planning Commission  (MS201632) |
| 63 | Zheng  et al, ^92^2020 | China | Parallel RCT | 120,mixed  cancer | Intervention:  61.0  Control: 60.4 | Acupoint application,60  Intensity: 4 week,2 per day,30 min per time;  The acupoint patch is primarily composed of metal particles such as TiO2, SiO2.  Acupoints:ST36,GV14, KI1 | Usual care, 60 | RPFS | Zhejiang Province Traditional Chinese Medicine Science and Technology Plan Project (2019ZB105) |
| 64 | Du  et al, ^93^2021 | China | Parallel RCT | 50,Intestinal cancer | Intervention:  55.6  Control: 61.8 | Acupuncture,26  Intensity: 2 weeks, 4 per week,30 min per time ;  Acupoints:CV4,CV6,ST36. | Usual care, 24 | PFS | China National Key Research and Development Plan (2018YFC1704102) |
| 65 | Han  et al, ^94^2021 | Korea | Parallel RCT | 96,Colorectal cancer | Intervention:  53.0  Control1: 56.4  Control2: 56.0 | Moxibustion,32  Intensity: 8 weeks, 2 per day,30 min per time;  Acupoints:CV8,CV12,ST36 ,LI4 | Usual care, 32  Sham moxibustion,32  using false acupoints | BFI | Korea Institute of Oriental Medicine  (K17121 and K18121). |
| 66 | Özdemir  et al, ^95^2021 | Turkey | Parallel RCT | 31,mixed cancer | Intervention:  69.7  Control: 70.3 | Acupressure,15  Intensity: 4 weeks, 2 per day,  18 min per time;  Acupoints:LI4,ST36,SP6. | Usual care, 16 | PFS | Erciyes University Scientific Research Projects  (TDK-2014-5025) |
| 67 | Song  et al, ^96^2021 | China | Parallel RCT | 80,Gastric cancer | Intervention:  55.9  Control: 56.8 | Moxibustion,40  Intensity: 6 weeks, 2 per day,30 min per time;  Acupoints:CV8,CV12,CV13 ,CV6,CV4. | Usual care, 40 | RPFS | Performance Incentive and Guidance Special Project of Scientific Research Institution, Chongqing Science and Technology Committee (cstc2018jxjl130008) |
| 68 | Chen  et al, ^97^2021 | China | Parallel RCT | 140,mixed cancer | Intervention:  62.0  Control: 62.0 | Moxibustion,154  Intensity: 1 weeks, once a day;  Acupoints:GV20,GV14,GV11,GV4,GV3. | Usual care, 78 | PFS | Special Project of Henan Provincial Administration of Traditional Chinese Medicine (2018ZY3019) |
| 69 | Chen  et al, ^98^2021 | China | Parallel RCT | 64,Lung  cancer | Intervention:  57.0  Control: 59.0 | Acupoint application,32  Intensity: week,once a day,2 h per time;  acupoint patch:Codonopsis18g, Fiveleaf Akebia Stem50g, Pinellia (Ban Xia) 10g, Aurantii Fructus 6g, Zhu Ru 10g, Aurantii Fructus Immaturus6g, White Atractylodes 15g, Poria 15g, Semen Sinapis Albae 3g, Licorice5g.  Acupoints:ST36,BL13,BL20,SP6 ,CV4. | Usual care, 32 | MFI | Guangdong Provincial Natural Science Foundation Project (2020A1515011176) |
| 70 | Liu  et al, ^99^2021 | China | Parallel RCT | 115,Gastric cancer | Intervention:  48.1  Control: 47.3 | Auricular acupressure,58  Intensity: 3 weeks, once a day,Stimulate each acupoint 2 min;  Ear Acupoints:shierzhichang,pi,wei,shenmen,pizhixia. | Usual care, 57 | EORTC QLQ-C30 | NA |
| 71 | Luan  et al, ^100^2021 | China | Parallel RCT | 60,Breast cancer | Intervention:  48.1  Control: 53.1 | Acupoint application,30  Intensity: 30 day,once a day,6 h per time;  acupoint patch:Bupleurum, turpentine, Hornwort, white art, jujube kernel, schisandra, albizia bark,Polygala, Gladiolus.  Acupoints:LR14 ,LR3 ,P6 ,SP6 ,KI1 | Usual care, 30 | RPFS | NA |
| 72 | Luo  et al, ^101^2021 | China | Parallel RCT | 67,Colorectal  cancer | Intervention:  54.5  Control: 55.4 | Moxibustion,34  Intensity: 9 weeks, Every other day,10 min each point;  Acupoints:CV4,CV8,ST36,BL20,BL23 | Usual care, 33 | RPFS | NA |
| 73 | Yang  et al, ^102^2021 | China | Parallel RCT | 61,Lung  cancer | Intervention:  47.9  Control: 47.6 | Moxibustion,29  Intensity: 9 weeks, once a day,20-30 min per time;  Acupoints:RN12,CV4,CV6,CV8,ST36,BL13, BL20. | Usual care, 32 | RPFS | Research Project of Guangxi Zhuang Autonomous Region Health Commission  (Z20190966)  Guangxi Graduate Education Innovation Program Funding Project  (YCSY2020091) |
| 74 | Zhao  et al, ^103^2021 | China | Parallel RCT | 99,Lung  cancer | Intervention:  61.9  Control: 61.9 | Moxibustion,49  Intensity: 4 weeks, once a day,30 min per time;  Acupoints:RN12,CV8,CV6,CV4. | Usual care, 50 | PFS | University level Research Project of Guangxi University of Traditional Chinese Medicine (2018QN026) |
| 75 | Liao  et al, ^104^2022 | China | Parallel RCT | 92,Breast  cancer | Intervention:  54.6  Control: 53.1 | Traditional Chinese exercises(Baduanjin),33  Intensity: 12 weeks,2 per week,90 min per time;  Detail:10-min warm-up, a 70-min Baduanjin form, and a 10-min cooldown. | Usual care, 35 | RPFS | Science and Technology Projects of Guangdong Province (20130325C)  Distinguishing Innovation Project of Department of Education of Guangdong Province (2015KTSCX080,2016KTSCX070)  Major International Cooperation Project of Department of Education of Guangdong Province  (2014WGJHZ005) |
| 76 | Xu  et al, ^105^2022 | China | Parallel RCT | 118,Lung  cancer | Intervention:  56.6  Control1: 55.9  Control2: 56.2 | Moxibustion,40  Intensity: 12 days ,once a day,30 min per time;  Acupoints:GV14,BL17,CV6,CV4  ,ST36.  . | Usual care, 39  Sham Moxibustion，39  Moxibustion was performed at a sham acupoint 1 cm far away from the true acupoints of the mild moxibus  tion group | RPFS | Shanghain Hospital Management Association (Q1902025)  Jiangsu University Clinical Medicine Technology Development Fund  (JIY2018008) |
| 77 | Yao  et al,^106^ 2022 | China | Parallel RCT | 72,Breast  cancer | Intervention:  45.3  Control: 48.6 | Traditional Chinese exercises(Tai Chi),36  Intensity: 8 weeks,2 per week,60 min per time;  Detail:including a 10-min warmup, 25–30 min of easy 8-form Yang style Tai chi practising, a 10-min break to rest during each session, and a 10-min cool-down | Usual care, 36 | BFI | Australian Government Research  Training Programme (RTP) scholarship, Australia |
| 78 | Ji  et al, ^107^2022 | China | Parallel RCT  Three arm | 88,Cancer of mouth | Intervention:  55.5  Control: 56.9 | Transcutaneous acupoint electrical stimulation,44  Intensity:12 days,2 per day,30 min per time;  Acupoints:BL20,BL23,LI4,ST30,SP6,RN4.  . | Sham acupressure,44  electrode pads were applied, and the electrode pads were fixed only to the stimulator leads without actual connection, so the patients did not receive actual TEAS stimulation. | RPFS | Shanghai's 2021 "Science and Technology Innovation Action Plan" Yangtze River Delta Science and Technology Innovation Community Project (21002411300) |
| 79 | Liu  et al, ^108^2022 | China | Parallel RCT | 160,mixed cancer | Intervention:  63.7  Control: 66.1 | Moxibustion,80  Intensity: 15 days ,every other day,35 min per time;  Acupoints:GV14,BL20,BL23 . | Usual care, 80 | RPFS | NA |
| 80 | Pei  et al, ^109^2022 | China | Parallel RCT | 93,mixed cancer | Intervention:  64.7  Control: 62.2 | Moxibustion,43  Intensity: 2 weeks ,once a day,10-15 min each Acupoint ;  Acupoints:ST36,CV6,CV4. | Acupressure,50  Intensity: 2 weeks, once a day, 2-3 min each Acupoint;  Acupoints:ST36,CV6,CV4. | BFI | National Natural Science Foundation of China (81703918) |
| 81 | Song  et al,^110^ 2022 | China | Parallel RCT | 71,Gynecological tumor | Intervention:  52.5  Control: 50.43 | Moxibustion,34  Intensity: 9 weekys ,once a day,6 per weeks,35 min per time;  Acupoints:CV8,CV6,CV4 . | Usual care, 37 | RPFS | Special Project for Internal Research of Guangdong Provincial Hospital of Traditional Chinese Medicine in China (YN2018HL06) |
| 82 | Xu  et al, ^111^2022 | China | Parallel RCT | 60,Hypopharyngeal carcinoma | Intervention:  58.7  Control: 58.5 | Moxibustion,30  Intensity: 2 weekys ,once a day,6 per weeks,20min per time;  Acupoints:ST36,SP6,CV4,BL23,CV6 . | Usual care, 30 | BFI | Shandong Province Traditional Chinese Medicine Science and Technology Development Plan (2019-0533) |
| 83 | Wen  et al, ^112^2022 | China | Parallel RCT | 75, Nasopharyngeal carcinoma | Intervention:  45.5  Control: 47.07 | Traditional Chinese exercises(Baduanjin),36  Intensity: 12 weeks,5 per week,40 min per time;  Detail:Each session lasted approximately 40 min, including a 5-min warmup before training, 25 min of Baduanjin exercise, and 10 min of posttraining cool-down | Usual care, 39 | MFI | NA |
| 84 | An  et al, ^113^  2022 | China | Parallel RCT | 78,Gastric cancer | Intervention:  53.9  Control: 55.5 | Auricular acupressure,37  Intensity: 6 days, 4-6 per day,3-5 min per time;  Ear Acupoints:  Benmen,pi,wei,gan,jiaogan,shenmen,pizhixia. | Usual care, 41 | CFS | NA |
| 85 | Zhang  et al, ^114^2023 | China | Parallel RCT | 68,mixed cancer | Intervention:  61.0  Control: 60.9 | Moxibustion,34  Intensity: 1 weekys ,every other day,3 per weeks,25-35 min per time;  Acupoints:The acupoints along the Governing Vessel. | Usual care, 34 | BFI | Medical Research Project of Mianyang Health and Family Planning Commission, 2018 Municipal Project (201802) |
| 86 | Wei  et al,^115^ 2022 | China | Parallel RCT | 107,breast cancer | Intervention:  45.5  Control: 47.07 | Traditional Chinese exercises(Baduanjin),52  Intensity: 12 weeks,5 per week,30 min per time;  Detail:The program began with stretching the joints, inhalation and exhalation for 2 min each, and two 12-min Baduanjin sessions, followed by 2 min of muscle relaxation  exercises. | Usual care, 55 | MFSI | Enhancement Project of Shanghai University of Traditional Chinese  Medicine  (2020HLXK07) |
| 87 | Huang  et al, ^116^2019 | China | Parallel RCT  Three arm | 120,mixed Cancer | Intervention:  58.2  Control: 59.1 | Transcutaneous acupoint electrical stimulation,60  Intensity:8 weeks,once a day,5 per week;30 min per time,10-100Hz,6-12mA;  Acupoints:CV4,CV6,SP10,CV8,ST36. | Auricular acupressure,60  Intensity: 8 weeks,once a day,5 per week,3-5 min each Acupoint;  Ear Acupoints:  Gan,pi,wei,shenmen,jiaogan | RPFS | NA |

Abbreviations: BFI, the Brief Fatigue Inventory; CFS, Cancer Fatigue Scale; EORTC QLQ-C30, the fatigue subscales of the European Organization for Research and Treatment of Cancer Quality of Life Questionnaire Core 30; FQ, Fatigue Questionnaire; FACIT-F, Functional Assessment of Chronic Illness Therapy–Fatigue; FSI, Fatigue Symptom Inventory; MFSI, Multidimensional Fatigue Symptom Inventory; MFI, Multidimensional Fatigue Inventory; NR, not report; RCT, randomized controlled trial; PRFS, Revised Piper Fatigue Scale; PFS, Piper Fatigue Scale; TCM, Traditional Chinese Medicine; TFRS, Tang Fatigue Rating Scale; USA, United States of America; UK, United Kingdom; VAS, Visual Analog Scale;

Usual care (standard or routine care): refers to the conventional medical treatment and practices that patients typically receive for their condition in a clinical setting. It includes standard procedures such as symptom management, medication, patient education, follow-up care, and other established protocols.
