# Supplementary material for: Efficacy of traditional Chinese medicine external therapy on cancer-related fatigue: a systematic review and network meta-analysis
Source: Front Oncol. 2026 Apr 22;16:1806355. doi: 10.3389/fonc.2026.1806355 (PMC13143725; doi:10.3389/fonc.2026.1806355)
Supplement: Supplementary file 11 [file Table5.docx]

**Supplementary table 5** CINeMA confidence.

| **Comparison** | **Number of studies** | **Within-study bias** | **Reporting bias** | **Indirectness** | **Imprecision** | **Heterogeneity** | **Incoherence** | **Confidence rating** |
| --- | --- | --- | --- | --- | --- | --- | --- | --- |
| 1:02 | 9 | Some concerns | High risk | No concerns | No concerns | Major concerns | No concerns | Very low |
| 1:03 | 5 | Some concerns | Low risk | Some concerns | No concerns | Major concerns | No concerns | Very low |
| 1:04 | 17 | Some concerns | High risk | Some concerns | No concerns | Major concerns | No concerns | Very low |
| 1:05 | 6 | Some concerns | Low risk | Some concerns | No concerns | Major concerns | No concerns | Very low |
| 1:06 | 25 | Some concerns | High risk | Some concerns | No concerns | No concerns | No concerns | Very low |
| 1:07 | 4 | Some concerns | Low risk | Some concerns | No concerns | Major concerns | Major concerns | Very low |
| 1:09 | 2 | Some concerns | High risk | No concerns | No concerns | No concerns | No concerns | Very low |
| 1:10 | 1 | Some concerns | Low risk | Some concerns | No concerns | Major concerns | No concerns | Very low |
| 1:11 | 5 | Some concerns | Low risk | Some concerns | No concerns | Major concerns | No concerns | Very low |
| 1:12 | 1 | Some concerns | Low risk | Some concerns | No concerns | Major concerns | No concerns | Very low |
| 1:13 | 2 | Some concerns | Low risk | Some concerns | No concerns | No concerns | No concerns | Low |
| 1:14 | 1 | Some concerns | Low risk | Some concerns | No concerns | Major concerns | No concerns | Very low |
| 1:15 | 5 | Some concerns | Low risk | Some concerns | No concerns | Major concerns | No concerns | Very low |
| 15:02 | 7 | No concerns | Low risk | Some concerns | No concerns | Major concerns | No concerns | Low |
| 15:03 | 2 | Some concerns | Low risk | Some concerns | No concerns | Major concerns | No concerns | Very low |
| 15:04 | 1 | Some concerns | Low risk | Some concerns | No concerns | Major concerns | No concerns | Very low |
| 15:05 | 2 | Some concerns | Low risk | Some concerns | No concerns | Major concerns | No concerns | Very low |
| 15:06 | 2 | Some concerns | Low risk | Some concerns | No concerns | Major concerns | No concerns | Very low |
| 15:08 | 3 | Some concerns | Low risk | Some concerns | No concerns | No concerns | No concerns | Low |
| 1:08 | 0 | Some concerns | Low risk | Some concerns | No concerns | No concerns | No concerns | Low |
| 15:07 | 0 | Some concerns | Low risk | Some concerns | No concerns | Major concerns | No concerns | Very low |
| 15:09 | 0 | Some concerns | Low risk | Some concerns | No concerns | Major concerns | No concerns | Very low |
| 10:15 | 0 | Some concerns | Low risk | Some concerns | No concerns | Major concerns | No concerns | Very low |
| 11:15 | 0 | Some concerns | Low risk | Some concerns | No concerns | Major concerns | No concerns | Very low |
| 12:15 | 0 | Some concerns | Low risk | Some concerns | Major concerns | No concerns | No concerns | Very low |
| 13:15 | 0 | Some concerns | Low risk | Some concerns | No concerns | No concerns | No concerns | Low |
| 14:15 | 0 | Some concerns | Low risk | Some concerns | Major concerns | No concerns | No concerns | Very low |
| Abbreviations: 1=Usual care; 2=Acupuncture; 3=Acupressure; 4=Traditional Chinese exercises; 5=Transcutaneous acupoint electrical stimulation;  6=Moxibustion; 7=Auricular acupressure; 8=Chinese medicine foot bath; 9=TCM emotional care; 10=Acupoint injection; 11=Acupoint application;  12=Auricular press needle; 13=warming needle; 14=Acupoint hot ironing; 15=placebo; | | | | | | | | |
