# Supplementary material for: Efficacy of traditional Chinese medicine external therapy on cancer-related fatigue: a systematic review and network meta-analysis
Source: Front Oncol. 2026 Apr 22;16:1806355. doi: 10.3389/fonc.2026.1806355 (PMC13143725; doi:10.3389/fonc.2026.1806355)
Supplement: Supplementary file 12 [file Table6.docx]

**Supplementary table 6** The data used for CINeMA assessments.

| **study** | **id** | **t** | **y** | **sd** | **n** | **rob** | **indirectness** |
| --- | --- | --- | --- | --- | --- | --- | --- |
| Molassiotis 2006 | 1 | 15 | 16.9 | 3 | 16 | 2 | 1 |
| Molassiotis 2006 | 1 | 2 | 14 | 2.4 | 16 | 2 | 1 |
| Molassiotis 2006 | 1 | 3 | 12.8 | 3.2 | 15 | 2 | 1 |
| Deng 2014 | 2 | 15 | 5.1 | 2 | 50 | 1 | 1 |
| Deng 2014 | 2 | 2 | 4.8 | 1.88 | 47 | 1 | 1 |
| Balk 2009 | 3 | 15 | 36.6 | 4.8 | 11 | 1 | 1 |
| Balk 2009 | 3 | 2 | 31.9 | 8.1 | 16 | 1 | 1 |
| Oh 2010 | 4 | 1 | 32.45 | 11.57 | 83 | 3 | 1 |
| Oh 2010 | 4 | 4 | 27.01 | 11.45 | 79 | 3 | 1 |
| Sun QZ 2011 | 5 | 1 | 6.17 | 0.11 | 30 | 2 | 2 |
| Sun QZ 2011 | 5 | 5 | 6.14 | 0.13 | 30 | 2 | 2 |
| Liu 2012 | 6 | 15 | 36.5 | 8.4 | 10 | 1 | 3 |
| Liu 2012 | 6 | 2 | 34.9 | 18.6 | 11 | 1 | 3 |
| Chen 2013 | 7 | 1 | 2.6 | 1.8 | 47 | 2 | 2 |
| Chen 2013 | 7 | 4 | 2.3 | 1.8 | 49 | 2 | 2 |
| Jiang MY 2013 | 8 | 1 | 4.19 | 1.28 | 30 | 2 | 2 |
| Jiang MY 2013 | 8 | 4 | 3.66 | 1.108 | 30 | 2 | 2 |
| Loh 2014 | 9 | 1 | 40.4 | 9.3 | 32 | 1 | 2 |
| Loh 2014 | 9 | 4 | 39.7 | 7.3 | 32 | 1 | 2 |
| Mao 2014 | 10 | 2 | 2.3 | 2.5 | 23 | 1 | 2 |
| Mao 2014 | 10 | 15 | 2.8 | 2.5 | 22 | 1 | 2 |
| Mao 2014 | 10 | 1 | 4 | 2.1 | 22 | 1 | 2 |
| Tang 2014 | 11 | 1 | 175.7 | 75 | 16 | 2 | 2 |
| Tang 2014 | 11 | 3 | 135.1 | 74.1 | 24 | 2 | 2 |
| Guo LY 2014 | 12 | 13 | 3.42 | 0.7 | 40 | 2 | 2 |
| Guo LY 2014 | 12 | 1 | 5.99 | 0.5 | 40 | 2 | 2 |
| Larkey 2015 | 13 | 4 | 2.1 | 1.34 | 44 | 1 | 2 |
| Larkey 2015 | 13 | 15 | 2.6 | 1.65 | 42 | 1 | 2 |
| Zhang RQ 2015 | 14 | 15 | 6.08 | 1.64 | 76 | 2 | 2 |
| Zhang RQ 2015 | 14 | 8 | 2.25 | 0.87 | 76 | 2 | 2 |
| Mao 2016 | 15 | 6 | 3.01 | 0.99 | 39 | 1 | 2 |
| Mao 2016 | 15 | 1 | 4.4 | 1.41 | 39 | 1 | 2 |
| Molassiotis 2021 | 16 | 4 | 26.78 | 6.58 | 78 | 3 | 1 |
| Molassiotis 2021 | 16 | 1 | 27.71 | 5.13 | 78 | 3 | 1 |
| Zick 2016 | 17 | 3 | 2.2 | 1.5 | 94 | 1 | 2 |
| Zick 2016 | 17 | 1 | 3.9 | 1.2 | 96 | 1 | 2 |
| Yeh 2016 | 18 | 4 | 5.2 | 1.06 | 51 | 3 | 2 |
| Yeh 2016 | 18 | 1 | 7.45 | 1.57 | 51 | 3 | 2 |
| Ding L 2016 | 19 | 9 | 13 | 4 | 43 | 2 | 2 |
| Ding L 2016 | 19 | 1 | 38 | 10 | 43 | 2 | 2 |
| He GJ 2016 | 20 | 4 | 3.9 | 0.65 | 31 | 3 | 2 |
| He GJ 2016 | 20 | 1 | 4.58 | 0.94 | 33 | 3 | 2 |
| Li SX 2016 | 21 | 2 | 7.51 | 3.35 | 40 | 2 | 1 |
| Li SX 2017 | 21 | 1 | 9.07 | 3.6 | 40 | 2 | 1 |
| Ma LX 2016 | 22 | 10 | 25.23 | 5.76 | 13 | 2 | 2 |
| Ma LX 2016 | 22 | 1 | 40.33 | 6.31 | 19 | 2 | 2 |
| Su Y 2016 | 23 | 2 | 4.15 | 0.89 | 30 | 2 | 1 |
| Su Y 2016 | 23 | 1 | 4.65 | 0.76 | 30 | 2 | 1 |
| Wu HY 2016 | 24 | 6 | 82.05 | 41.22 | 60 | 2 | 2 |
| Wu HY 2016 | 24 | 1 | 109.37 | 40.06 | 60 | 2 | 2 |
| Zhang X2016 | 25 | 6 | 46.46 | 17.36 | 22 | 2 | 2 |
| Zhang X2016 | 25 | 1 | 62.12 | 9.49 | 22 | 2 | 2 |
| chen 2017 | 26 | 2 | 4.5 | 0.3 | 14 | 1 | 2 |
| chen 2017 | 26 | 15 | 7.1 | 0.3 | 14 | 1 | 2 |
| Chuang 2017 | 27 | 4 | 17.36 | 18.03 | 48 | 1 | 2 |
| Chuang 2017 | 27 | 1 | 78.23 | 15.37 | 48 | 1 | 2 |
| Hou 2017 | 28 | 5 | 2.06 | 0.9 | 57 | 1 | 2 |
| Hou 2017 | 28 | 1 | 3 | 1.29 | 56 | 1 | 2 |
| McQuade 2016 | 29 | 4 | 1.81 | 0.35 | 21 | 2 | 2 |
| McQuade 2016 | 29 | 1 | 2.59 | 0.39 | 24 | 2 | 2 |
| Wu YF 2017 | 30 | 5 | 2.06 | 0.9 | 57 | 2 | 2 |
| Wu YF 2017 | 30 | 15 | 2.8 | 1.34 | 49 | 2 | 2 |
| Wu YF 2017 | 30 | 1 | 3 | 1.24 | 56 | 2 | 2 |
| Xu XZ 2017 | 31 | 6 | 2.65 | 1.53 | 40 | 2 | 1 |
| Xu XZ 2017 | 31 | 1 | 4.23 | 1.98 | 40 | 2 | 1 |
| Yu MW 2017 | 32 | 2 | 5.38 | 1.35 | 34 | 1 | 2 |
| Yu MW 2017 | 32 | 15 | 5.6 | 1.63 | 30 | 1 | 2 |
| Yuan M 2017 | 33 | 6 | 3.7 | 1.3 | 32 | 2 | 2 |
| Yuan M 2017 | 33 | 1 | 5.02 | 1.82 | 32 | 2 | 2 |
| Chen SJ 2018 | 34 | 9 | 16.41 | 2.82 | 32 | 2 | 1 |
| Chen SJ 2018 | 34 | 1 | 20.09 | 3.42 | 32 | 2 | 1 |
| Deng XY 2018 | 35 | 2 | 36.66 | 16.02 | 30 | 2 | 2 |
| Deng XY 2018 | 35 | 1 | 59.62 | 14.44 | 30 | 2 | 2 |
| Han B 2018 | 36 | 11 | 2.63 | 0.43 | 60 | 2 | 1 |
| Han B 2018 | 36 | 1 | 4.17 | 0.67 | 60 | 2 | 1 |
| Jiang SP 2018 | 37 | 6 | 2.94 | 0.61 | 35 | 2 | 2 |
| Jiang SP 2018 | 37 | 1 | 3.72 | 0.75 | 35 | 2 | 2 |
| Liang H 2018 | 38 | 6 | 2.6 | 0.4 | 34 | 2 | 2 |
| Liang H 2018 | 38 | 1 | 3.6 | 0.5 | 29 | 2 | 2 |
| Shao J 2018 | 39 | 12 | 4.43 | 1.13 | 40 | 2 | 2 |
| Shao J 2018 | 39 | 1 | 5.04 | 1.52 | 40 | 2 | 2 |
| Xu MN 2018 | 40 | 6 | 2.2 | 0.894 | 20 | 2 | 2 |
| Xu MN 2018 | 40 | 1 | 4 | 0.795 | 20 | 2 | 2 |
| Zhang Y 2018 | 41 | 7 | 20.57 | 3.87 | 28 | 2 | 2 |
| Zhang Y 2018 | 41 | 1 | 26.4 | 3.31 | 30 | 2 | 2 |
| Khanghah 2019 | 42 | 3 | 6.03 | 1.69 | 30 | 2 | 1 |
| Khanghah 2019 | 42 | 15 | 6.2 | 1.92 | 30 | 2 | 1 |
| Khanghah 2019 | 42 | 1 | 7.23 | 1.46 | 30 | 2 | 1 |
| Lu 2019 | 43 | 4 | 2.7 | 2.1 | 43 | 2 | 2 |
| Lu 2019 | 43 | 1 | 4.1 | 1.9 | 44 | 2 | 2 |
| Chen D 2019 | 44 | 6 | 2.75 | 0.57 | 30 | 2 | 1 |
| Chen D 2019 | 44 | 1 | 3.94 | 0.79 | 30 | 2 | 1 |
| Chen J 2019 | 45 | 6 | 5.05 | 1.8 | 30 | 2 | 1 |
| Chen J 2019 | 45 | 1 | 5.92 | 1.13 | 30 | 2 | 1 |
| Ge DH 2019 | 46 | 4 | 4.48 | 0.47 | 39 | 2 | 1 |
| Ge DH 2019 | 46 | 1 | 5.44 | 0.67 | 39 | 2 | 1 |
| Han Q 2019 | 47 | 1 | 5.13 | 1.73 | 23 | 2 | 2 |
| Han Q 2019 | 47 | 4 | 2.95 | 0.88 | 21 | 2 | 2 |
| He PS 2019 | 48 | 11 | 3.08 | 1.1 | 26 | 3 | 2 |
| He PS 2019 | 48 | 1 | 3.97 | 1.11 | 25 | 3 | 2 |
| Wang LX 2019 | 49 | 8 | 2.11 | 0.68 | 36 | 2 | 2 |
| Wang LX 2019 | 49 | 15 | 3.16 | 0.51 | 36 | 2 | 2 |
| Wang XQ 2019 | 50 | 8 | 2.55 | 0.67 | 34 | 2 | 2 |
| Wang XQ 2019 | 50 | 15 | 4.12 | 0.75 | 30 | 2 | 2 |
| Wang XY 2019 | 51 | 14 | 44.44 | 24.69 | 30 | 2 | 2 |
| Wang XY 2019 | 51 | 1 | 55.56 | 8.234 | 30 | 2 | 2 |
| Xia WM 2019 | 52 | 6 | 32.04 | 8.4 | 37 | 2 | 2 |
| Xia WM 2019 | 52 | 1 | 38.46 | 9.37 | 37 | 2 | 2 |
| Xu DM 2019 | 53 | 5 | 48.6 | 10.2 | 41 | 2 | 2 |
| Xu DM 2019 | 53 | 1 | 68.4 | 13.6 | 41 | 2 | 2 |
| Zhang ZX 2019 | 54 | 6 | 17.68 | 1.96 | 38 | 2 | 2 |
| Zhang ZX 2019 | 54 | 1 | 24.37 | 2.69 | 40 | 2 | 2 |
| Nakano 2020 | 55 | 5 | 35.6 | 22.6 | 24 | 2 | 3 |
| Nakano 2020 | 55 | 1 | 41 | 22.1 | 24 | 2 | 3 |
| Tao LX 2020 | 56 | 2 | 4.17 | 1.14 | 32 | 2 | 1 |
| Tao LX 2020 | 56 | 1 | 5.17 | 1.29 | 32 | 2 | 1 |
| Li Y 2020 | 57 | 5 | 3.81 | 0.66 | 55 | 2 | 1 |
| Li Y 2020 | 57 | 1 | 4.44 | 0.85 | 53 | 2 | 1 |
| Lu N 2020 | 58 | 6 | 33.7 | 5.22 | 32 | 2 | 2 |
| Lu N 2020 | 58 | 1 | 35.04 | 5.7 | 31 | 2 | 2 |
| Ni P 2020 | 59 | 1 | 4.3 | 0.8 | 37 | 2 | 2 |
| Ni P 2020 | 59 | 7 | 2.9 | 0.6 | 37 | 2 | 2 |
| Qin P 2019 | 60 | 1 | 85.89 | 18.03 | 31 | 2 | 1 |
| Qin P 2019 | 60 | 2 | 72.17 | 12.06 | 36 | 2 | 1 |
| Su J 2020 | 61 | 6 | 2.51 | 0.76 | 56 | 2 | 2 |
| Su J 2020 | 61 | 1 | 3.7 | 0.84 | 55 | 2 | 2 |
| Xu T 2020 | 62 | 4 | 30.6 | 7.6 | 46 | 2 | 2 |
| Xu T 2020 | 62 | 1 | 56.3 | 8.4 | 46 | 2 | 2 |
| Zheng XQ 2020 | 63 | 11 | 2.45 | 0.41 | 60 | 2 | 1 |
| Zheng XQ 2020 | 63 | 1 | 3.59 | 0.5 | 60 | 2 | 1 |
| du 2021 | 64 | 2 | 3.4 | 0.97 | 26 | 3 | 2 |
| du 2021 | 64 | 1 | 4.02 | 1.51 | 24 | 3 | 2 |
| Han 2021 | 65 | 6 | 3.83 | 2.18 | 32 | 1 | 2 |
| Han 2021 | 65 | 15 | 4.98 | 2.3 | 32 | 1 | 2 |
| Han 2021 | 65 | 1 | 6 | 2.14 | 32 | 1 | 2 |
| ?zdemir 2021 | 66 | 3 | 3.21 | 2.07 | 15 | 2 | 2 |
| ?zdemir 2021 | 66 | 1 | 5.49 | 1.89 | 16 | 2 | 2 |
| Song 2021 | 67 | 6 | 3.95 | 0.55 | 40 | 2 | 2 |
| Song 2021 | 67 | 1 | 5.54 | 0.75 | 40 | 2 | 2 |
| Chen L 2021 | 68 | 6 | 5.76 | 0.5 | 78 | 2 | 1 |
| Chen L 2021 | 68 | 1 | 5.94 | 0.48 | 154 | 2 | 1 |
| Chen ZR 2021 | 69 | 11 | 49.56 | 6.31 | 32 | 2 | 2 |
| Chen ZR 2021 | 69 | 1 | 53.28 | 6.48 | 32 | 2 | 2 |
| Liu H 2021 | 70 | 7 | 13.21 | 6.92 | 57 | 3 | 2 |
| Liu H 2021 | 70 | 1 | 17.95 | 7.28 | 58 | 3 | 2 |
| Luan YF 2021 | 71 | 11 | 3.63 | 0.78 | 30 | 2 | 2 |
| Luan YF 2021 | 71 | 1 | 4.69 | 0.9 | 30 | 2 | 2 |
| Luo 2021 | 72 | 6 | 4.21 | 0.69 | 34 | 2 | 2 |
| Luo 2021 | 72 | 1 | 5.8 | 0.51 | 33 | 2 | 2 |
| Yang H 2021 | 73 | 6 | 3.06 | 0.23 | 29 | 2 | 2 |
| Yang H 2021 | 73 | 1 | 4.84 | 0.24 | 32 | 2 | 2 |
| Zhao YL 2021 | 74 | 6 | 3.37 | 0.52 | 49 | 2 | 2 |
| Zhao YL 2021 | 74 | 1 | 5.76 | 0.28 | 50 | 2 | 2 |
| liao 2022 | 75 | 4 | 14.24 | 12.47 | 33 | 1 | 2 |
| liao 2022 | 75 | 1 | 27.49 | 22.16 | 35 | 1 | 2 |
| xu 2022 | 76 | 6 | 1.84 | 0.71 | 42 | 2 | 2 |
| xu 2022 | 76 | 15 | 2.13 | 0.61 | 42 | 2 | 2 |
| xu 2022 | 76 | 1 | 2.23 | 0.77 | 42 | 2 | 2 |
| yao 2022 | 77 | 4 | 3.53 | 2.02 | 36 | 3 | 2 |
| yao 2022 | 77 | 1 | 5.2 | 1.97 | 36 | 3 | 2 |
| Ji PP 2022 | 78 | 5 | 6 | 0.71 | 44 | 2 | 2 |
| Ji PP 2022 | 78 | 15 | 6.44 | 0.42 | 44 | 2 | 2 |
| Liu SH 2022 | 79 | 6 | 3.22 | 1.69 | 80 | 2 | 1 |
| Liu SH 2022 | 79 | 1 | 5.11 | 1.38 | 80 | 2 | 1 |
| Pei YX 2022 | 80 | 6 | 2.2 | 0.6 | 43 | 2 | 1 |
| Pei YX 2022 | 80 | 3 | 2.5 | 0.5 | 50 | 2 | 1 |
| Song JT 2022 | 81 | 6 | 4.63 | 0.49 | 34 | 2 | 2 |
| Song JT 2022 | 81 | 1 | 6.26 | 0.42 | 37 | 2 | 2 |
| Xu J 2022 | 82 | 6 | 3.52 | 0.47 | 30 | 2 | 2 |
| Xu J 2022 | 82 | 1 | 6.39 | 0.51 | 30 | 2 | 2 |
| Wen 2022 | 83 | 4 | 48.08 | 10.52 | 36 | 2 | 2 |
| Wen 2022 | 83 | 1 | 54.95 | 10.82 | 39 | 2 | 2 |
| An HY 2022 | 84 | 7 | 28.95 | 4.29 | 36 | 2 | 2 |
| An HY 2022 | 84 | 1 | 31.27 | 4.08 | 41 | 2 | 2 |
| Zhang YQ 2022 | 85 | 6 | 4.01 | 1.06 | 34 | 2 | 1 |
| Zhang YQ 2022 | 85 | 1 | 8.32 | 1.9 | 34 | 2 | 1 |
| Wei 2022 | 86 | 4 | 1.95 | 0.85 | 35 | 2 | 2 |
| Wei 2022 | 86 | 1 | 2.99 | 1.51 | 35 | 2 | 2 |
| Huang F 2023 | 87 | 5 | 3.19 | 1.23 | 60 | 2 | 1 |
| Huang F 2023 | 87 | 7 | 4.28 | 1.85 | 60 | 2 | 1 |
